# Comparison of Phosphoribosyl Ubiquitin Probes Targeting *Legionella* Dup Enzymes

**DOI:** 10.1021/acs.bioconjchem.4c00541

**Published:** 2025-02-17

**Authors:** Max S. Kloet, Rishov Mukhopadhyay, Rukmini Mukherjee, Mohit Misra, Cami M. P. Talavera Ormeño, Rayman T. N. Tjokrodirijo, Paul J. Hensbergen, Peter A. van Veelen, Ivan Đikić, Aysegul Sapmaz, Gerbrand J. van der Heden van Noort

**Affiliations:** † Department of Cell and Chemical Biology, Leiden University Medical Centre, 2333 ZCCLeiden, The Netherlands; ‡ Buchmann Institute for Molecular Life Sciences, Goethe University, 60348Frankfurt, Germany; § Center for Proteomics and Metabolomics, Leiden University Medical Center, 2333 ZCLeiden, The Netherlands

## Abstract

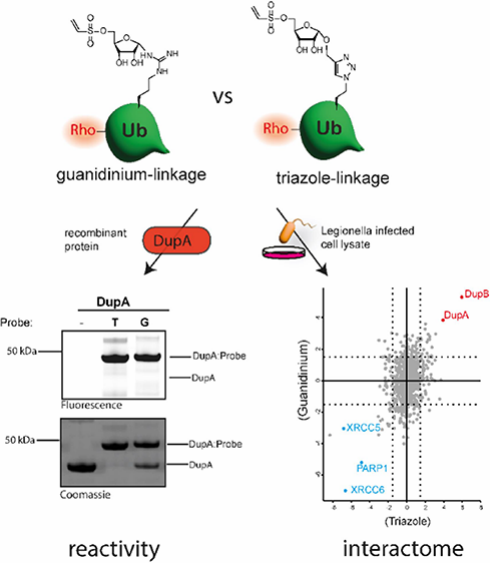

In order to effectively replicate within a host cell, the *Legionella pneumophila* bacterium secretes effector enzymes into the cytoplasm in order to manipulate cellular host pathways including host ubiquitination. Some of these effectors, the so-called SidE-family, mediate noncanonical phosphoribosyl serine ubiquitination (PR-ubiquitination) of host substrate proteins, contributing to the recruitment of ER-remodeling proteins and the formation of a *Legionella*-containing vacuole, which is crucial in the early stages of bacterial infection. PR-ubiquitination is a dynamic process that is reversed by other *Legionella* effectors called deubiquitinases for PR-ubiquitination (Dups). We recently discovered a reactive allosteric cysteine in close proximity to the catalytic triad of DupA, which can be exploited as a target for covalent probe development. We here report on the synthesis of vinyl-sulfonate and fluoro-sulfonate warhead-containing phosphoribosyl ubiquitin probes, where the Arg42 position of ubiquitin is linked to the C1 of ribose via a native guanidinium group, and compare them to triazole-linked probes. In vitro tests on recombinant DupA and SdeA_PDE_ revealed that these probes are able to capture the enzymes covalently. In a pull-down proteomics experiment, DupA and DupB enzymes are enriched from *Legionella*-infected cell lysates, highlighting the potential of native Arg-riboside linked probes to capture *Legionella* effector enzymes in a complex proteome.

## Introduction

**Figure 1:**
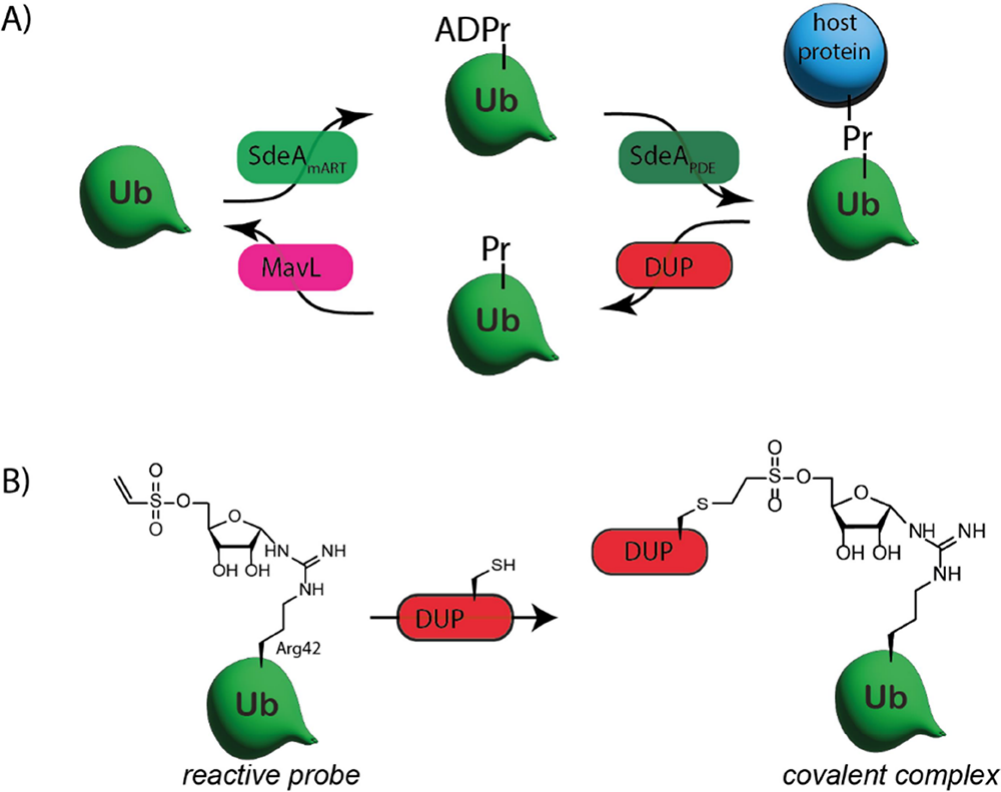
(A) Schematic representation of the PR-ubiquitination mechanism *Legionella* utilizes to control the host’s ubiquitination system. (B) Expected chemical trapping of the near-active site allosteric Cys196 in DupA by our substrate mimicking probe possessing the native arginine linkage.

The *Legionella pneumophila* bacterium, the causative agent of Legionnaires disease, releases a multitude of effector enzymes into the host cell it invades. Among these, a unique set of enzymes ubiquitinates host substrates in a highly unconventional manner. This SidE effector family, which comprises SdeA, SdeB, SdeC, and SidE, are large (>150 kDa) multidomain proteins that initiate a multistep cascade. This process (Figure 1A) begins with ADPribosylation of the host ubiquitin on Arg42 by the monoADPribosyl transferase (mART) domain, at the expense of NAD^+^.^1,2^ After being processed by the mART domain, Arg42 ADP-ribosylated ubiquitin is transferred to the SidE phosphodiesterase (PDE) domain. This domain mediates conjugation to a serine residue in a host substrate protein while expelling AMP. In contrast to canonical Lys-ubiquitination, these combined SidE activities lead to a phosphoribosyl (PR) bridge between the substrate and Arg42 of ubiquitin. In this manner, predominantly ER regulatory and Golgi proteins are modified by the bacterium.^3,4^*Legionella* manipulates the host’s ubiquitin system to gain local control and direct PR-ubiquitinated substrates to the *Legionella*-containing vacuole (LCV) to establish and maintain an environment conducive to bacterial replication. This strategy is crucial for *Legionella’s* ability to proliferate within host cells and evade the immune response. Without the SidE effectors, which play a key role in this process, bacterial replication is significantly impaired.^1,5,6^ This SidE PR-Ub ligase activity is highly dynamic and counteracted and regulated by, among others, deubiquitinases for phosphoribosyl ubiquitination (Dups).^3,7^ These Dups (DupA and DupB) are composed solely of a PDE-like domain and cleave the phosphomonoester of serine PR-ubiquitinated proteins to liberate the native host protein. Concomitantly, this process produces phosphoribosyl ubiquitin, a molecule that is thought to be incompatible with subsequent conventional host ubiquitination and is speculated to act as an autophagy blockade.^3,7,8^ Moreover, the combined SidE/Dup activities have been shown to regulate Golgi morphology and ER fragmentation to establish and maintain the LCV, highlighting their critical roles in infection.^9^ From a therapeutic perspective, the *Legionella* effectors that coordinate PR-ubiquitination could be interesting candidates for drug development, given their crucial and unique roles in the onset of *Legionella* infection. The development of tools to study the molecular mechanism of action of these enzyme families hence forms the basis of such endeavors.

The PDE domains of SidE-class PR-Ub ligase effectors, along with the hydrolases DupA and DupB, share a Glu-His-His active site triad, which is crucial for catalyzing the conjugation or deconjugation of their respective substrates. Interestingly, both Dup enzymes contain a reactive allosteric cysteine near the catalytic triad, which can be exploited to covalently trap the protein and facilitate the enrichment of Dups from *Legionella*-infected lysates. Based on our recently reported methodology to prepare native Ub^ADPr^,^10^ linked via the guanidinium side chain of arginine, and protease-stable covalent probes to capture Dups,^11,12^ we set out to develop a novel set of covalent probes more closely resembling the native substrates of these *Legionella* enzymes by fusing both methods (Figure 1B). Our previous works show that the *Legionella* effectors DupA and SdeA tolerate the triazole bond formed via click chemistry as an Arg42 mimic, and the application of such probes, equipped with a reactive warhead, to effectively label recombinant DupA highlights their power.^12−14^ However, we also reported on a reduction in the K_D_ of triazole-linked Ub^ADPr^ compared to the native guanidinium-linked Ub^ADPr^.^14^ Consequently, we envision that native guanidinium-linked Ub^Pr^-mimicking probes that have a structure resembling the native substrates more closely might be more effective. Potentially restoring the more flexible and H-bond forming guanidinium linkage instead of the rigid, uncharged triazole linkage could enhance Dup recognition.

We report here on the development of a chemical methodology to synthesize native guanidinium-linked Ub-phoshoribose conjugates as covalent probes and investigate their reactivity on recombinant *Legionella* DupA and SdeA PDE-domain proteins in gel-based assays. Subsequently, a pulldown from lysates of *Legionella*-infected HEK293T cells is performed, and the proteomic analyses are evaluated and compared to the previously reported analoguous triazole probes. The guanidinium Ub-phosphoribose probes effectively bind to *Legionella* enzymes both at the recombinant protein level and in *Legionella*-infected lysates, thereby expanding the PR-ubiquitination toolbox to study *Legionella* effectors.

## Results & Discussion

### Synthesis of Natively Linked Arginine Probes

**Scheme 1:**
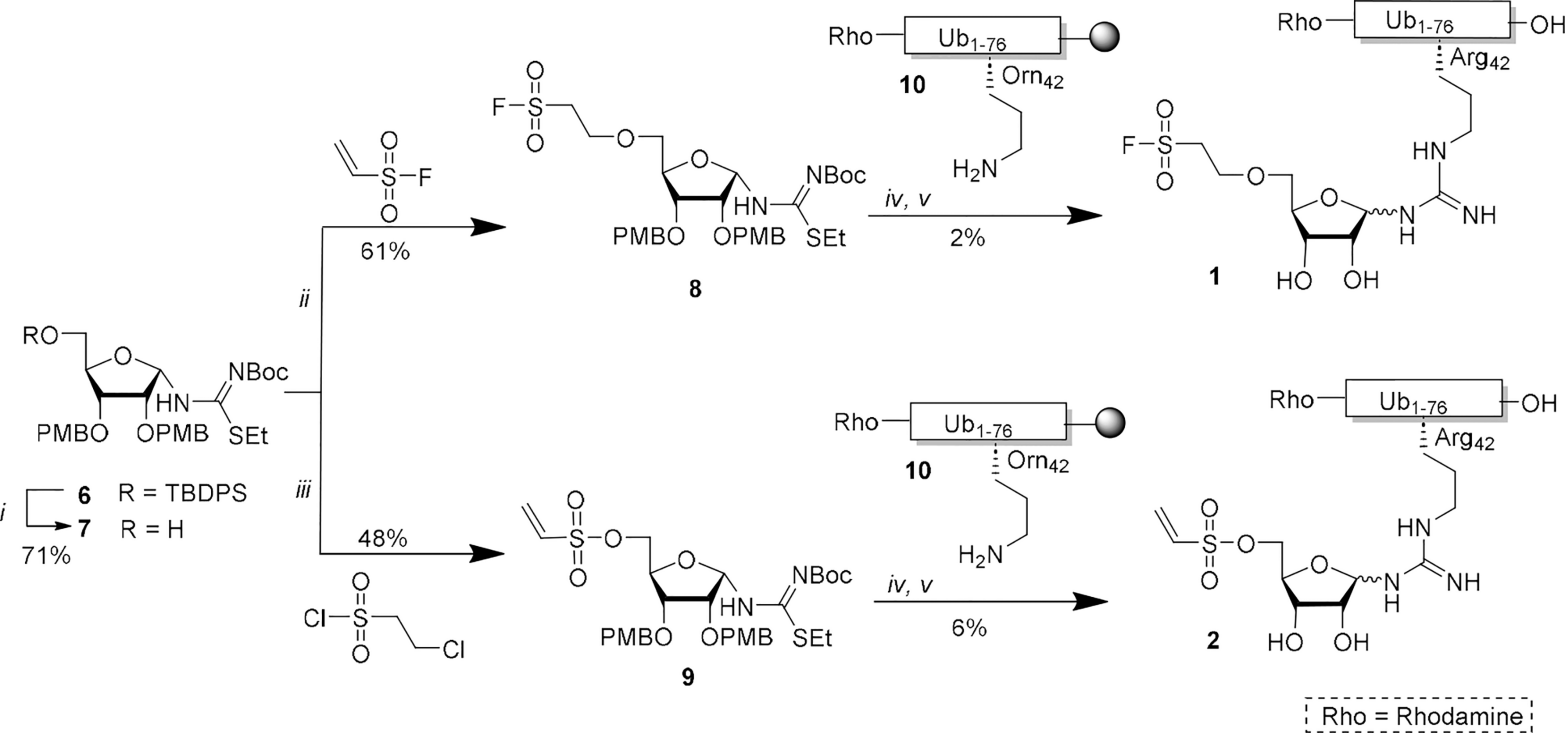
Synthesis of Fluoro-sulfonate and Vinyl-sulfonate Ub Probes **1** and **2** Reagents and conditions: (i) TBAF, THF, rt; (ii) vinyl sulfonyl fluoride, Et_3_N, DCM, rt, (iii) 2-chloroethane sulfonylchloride, Et_3_N, DCM, 0 °C → rt, (iv) Rho-Ub_1-76_ (Arg42Orn) **10**, AgNO_3_, Et_3_N, DMF, rt; (v) TFA:TIS:H_2_O:phenol (90.5:2:5:2.5, v/v/v/v).

Starting from protected isothiourea N-riboside^10^**6**, the TBDPS group was removed with TBAF to yield **7** in 71% (Scheme 1). Then, ethene sulfonyl fluoride and triethylamine were employed to effectively install the fluoro-sulfonate warhead **8** in 61%. To install the vinyl-sulfonate warhead on **7**, 2-chloroethane sulfonyl chloride and triethylamine were applied, and **9** was isolated in 48%. Subsequently, the warhead-equipped ribosides **8** and **9** were coupled to the free amine of ornithine on position 42 in ubiquitin **1** on-resin under silver-ion mediation to form the native guanidinium linkage. Acid-mediated deprotection of the conjugates proceeded uneventfully and yielded **1** and **2** in 2 and 6% yields after HPLC purification, calculated from the initial loading of the resin, respectively. Importantly, the fluoro-sulfonate and vinyl-sulfonate warheads as well as the glycosidic bond linking ubiquitin to the riboside remained intact during the acid-mediated deprotection. Based on the results described earlier, the acid-mediated release from the resin is expected to induce anomerization of the glycosidic bond to result in the isolation of the natively linked probes as α- and β- anomeric mixtures (6:4, α:β).^10^ This is in contrast to the method used for the construction of the triazole-linked probes using CuAAC, where no harsh acidic conditions are used in the preparation of the probes, and hence anomerization is unlikely to occur. Additionally, the conjugated full-length ubiquitin is equipped with a rhodamine fluorophore on the N-terminus, resulting in two fluorescently labeled covalent probes.

### Assessment of Probe Reactivity on Recombinant DupA and SdeA_PDE_

**Figure 2:**
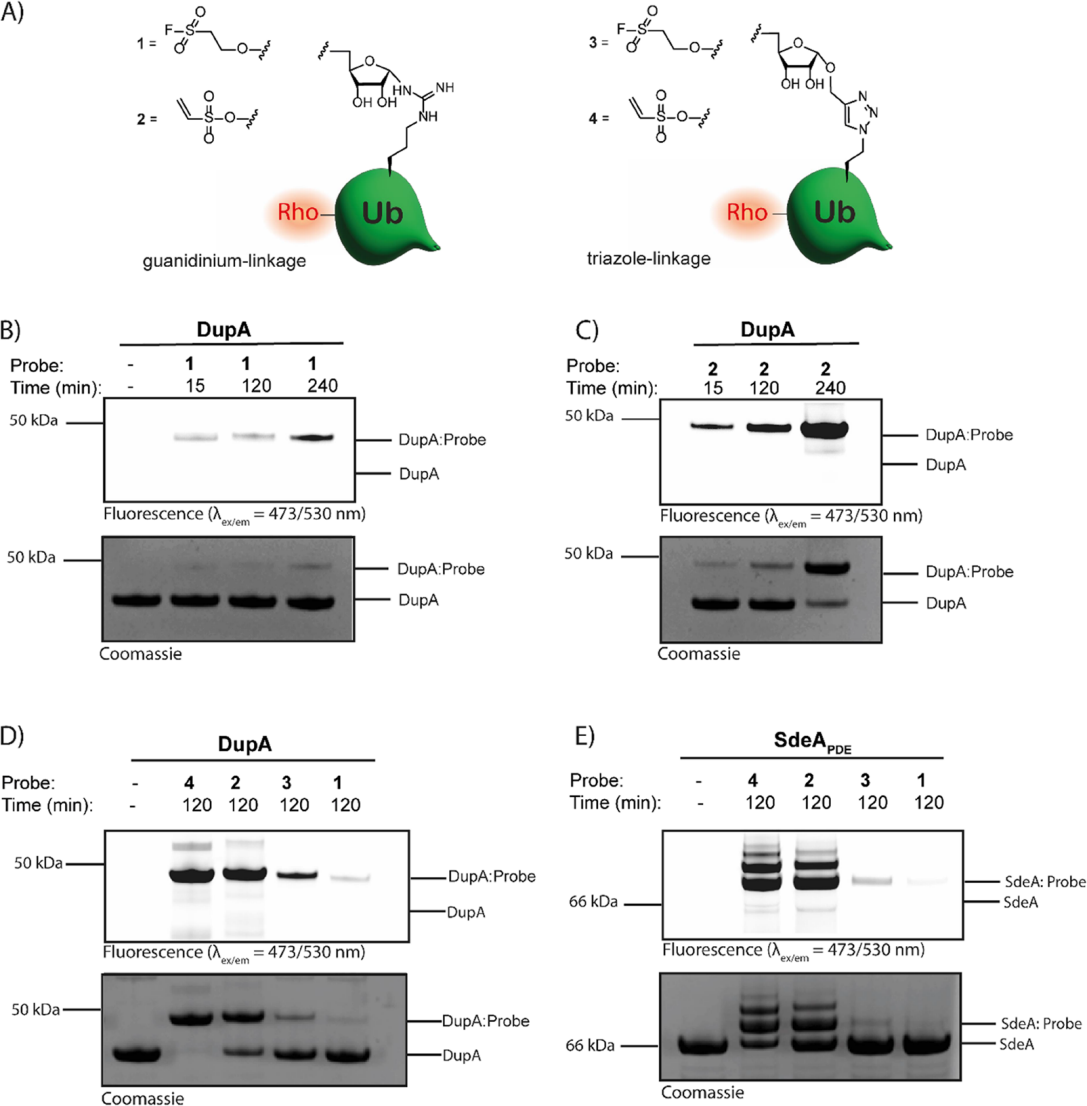
Comparison of probe reactivity toward recombinant DupA and SdeA_PDE_. (A) Structure of guanidinium-linked probes **1** and **2** and triazole-linked probes **3** and **4**. (B) Covalent complex formation between fluoro-sulfonate probe **1** and DupA WT at the indicated time points as analyzed by SDS-PAGE, Coomassie stain (upper panel), and fluorescent scan (bottom panel). (C) Covalent complex formation between vinyl-sulfonate probe **2** and DupA WT at the indicated time points as analyzed by SDS-PAGE. (D) Comparison of fluoro-sulfonate probes **1** and **3** and vinyl-sulfonate probes **2** and **4** incubated with DupA WT for 2 h and complex formation analyzed by SDS-PAGE. (E) Comparison of fluoro-sulfonate probes **1** and **3** and vinyl-sulfonate probes **2** and **4** incubated with SdeA_PDE_ WT for 2 h and complex formation analyzed by SDS-PAGE.

The reactivity of the natively linked fluorescently labeled probes **1** and **2** toward DupA WT was examined by visualizing DupA:probe complex formation over time using SDS-PAGE analysis. Moderate labeling of DupA WT by fluoro-sulfonate probe **1** at the later time points (Figure 2B) was observed. Conversely, vinyl-sulfonate probe **2** already shows efficient complex formation after 15 min, reaching near complete conversion after 4 h of incubation with DupA WT (Figure 2C). This indicates that both probes are indeed recognized by DupA and able to form a covalent bond, although with different kinetics. Subsequently, the effect of the protein-ribose linkage, either guanidinium in probes **1** and **2**, or triazole in earlier reported probes **3** or **4**, on DupA labeling was investigated at the intermediate 2 h time point (Figure 2D). Triazole probes **3** and **4** gave a higher level of DupA labeling than their natively linked counterpart probes **1** and **2**. The reduced reactivity of the guanidinium-linked probes can potentially be attributed to the aforementioned anomerization, precluding part of the probe from reacting due to a misorientation of the riboside. We also conducted a labeling experiment with the more reactive vinyl-sulfonate probes **2** and **4** on the DupA Cys196Ala mutant, and a notable observation is the significant labeling of both probes, reaching more than 50% conversion after 2 h (Supplemental Figure 1). We previously established the site of covalent reactivity toward probe **4** to Cys196 using tandem mass spectrometry, crystal structure analysis, and mutational analyses.^12^ The reactivity toward the DupA Cys196Ala mutant however suggests that both probes **2** and **4** may potentially react with another nucleophilic amino acid when Cys196 is mutated, although this reaction proceeds at a slower rate. We hence performed tryptic digestion, followed by tandem MS analysis of the formed complexes by probe **4** with DupA Cys196Ala and DupA WT, respectively. The tryptic peptides of the DupA Cys196Ala mutant clearly identified the cross-linking of the Ub probe to Lys197, indicating that upon mutation of Cys196, the adjacent lysine residue is able to react with the vinyl-sulfonate warhead (Supplemental Figure 2). Upon closer inspection, we also found probe **4** to react with Lys197 in DupA WT in addition to the previously reported Cys196 (Supplemental Figure 3).

The probes were tested next on their potential to react with SdeA_PDE_, which is one of the four SidE ligases for PR-ubiquitination crucial in *Legionella* infection. The PDE domains of SidE ligases have high structural homology to the Dups, although the allosteric cysteine we target in Dups (Cys196) is not conserved in the SidEs. SidE PDE domains, however, do contain other cysteine residues (Cys560, Cys567) close to the active site that might be labeled using our probes. Indeed reactivity of SdeA_PDE_ toward the probe was observed, and labeling with the vinyl-sulfonate warhead seemed superior over the fluoro-sulfonate warhead. In addition and comparable to DupA, the triazole-linked probe **4** proved to be the most efficient (Figure 2E). Notably, next to single also, double labeling of SdeA_PDE_ (222–592) by triazole-linked probe **4** and guanidinium-linked probe **2** was observed, which might be off-target reactivity to a nucleophilic side chain elsewhere in the protein. In the case of the fluoro-sulfonate probes **1** and **3**, only moderate labeling of SdeA_PDE_ to the triazole-linked analogue **3** was observed, and traces of covalent attachment to guanidinium-linked **1** were visible in line with reactivity of these probes to DupA.

### Guanidinium-Linked Vinyl-sulfonate Probe Is Able To Enrich Dups from *Legionella*-Infected HEK293T Cell Lysate

**Figure 3:**
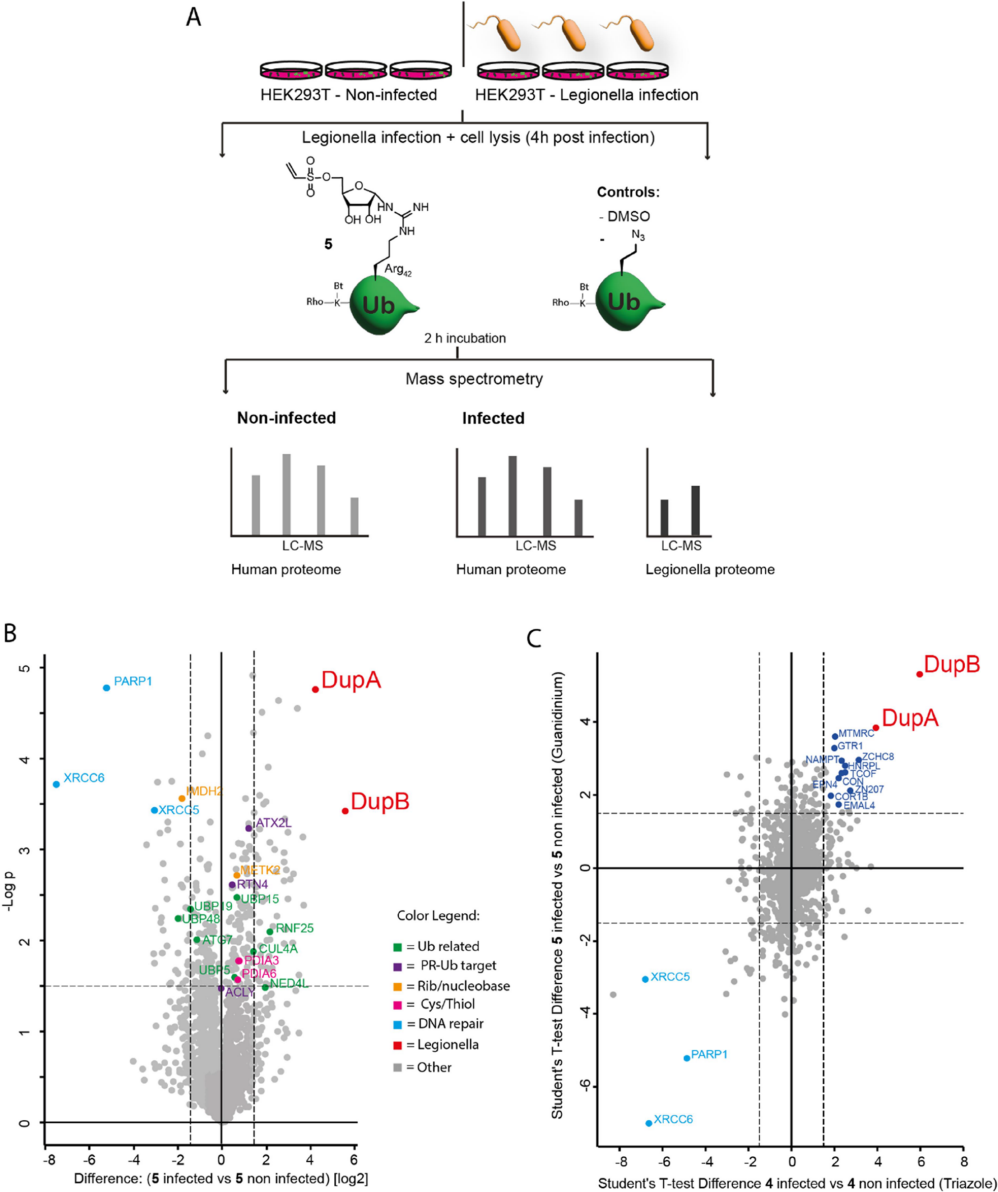
Proteomic assessment of pulldown by the guanidinium-linked vinyl-sulfonate probe **5** from noninfected HEK293T cells or cells infected with *Legionella*. (A) Schematic representation of the workflow applied in mass spectrometry-based proteomics. The lysates of two sample groups; infected with *Legionella* and noninfected, were prepared in triplicate. (B) Volcano plot depicting proteins enriched by probe **5** comparing the infected sample group to the noninfected sample group. (C) Student’s *t-*test analysis comparing enrichment by probe **5** between the infected and noninfected cells to enrichment by triazole probe **4** in the infected and noninfected sample groups. For every plot holds: black dashed lines correspond to the thresholds: log_2_ ratio ≥1.5; *p*-value ≤0.05. A color code legend is provided for the clustered proteins. The *Legionella* enzymes DupA and DupB are highlighted in red.

Given the swift and effective labeling of recombinant DupA by the natively linked probe **2**, we set out to validate the potential of the probe to monitor Dups in the lysate of human HEK293T cells infected with *Legionella pneumophila* and compare the results to the triazole-linked variant **4**. To facilitate this pull-down proteomic experiment, we prepared a dual rhodamine and biotinylated vinyl-sulfonate probe **5**. Both noninfected HEK293T cells and cells infected with *Legionella pneumophila* were lysed 4 h postinfection as the optimal time point for detecting Dups.^3^ The lysates were subjected to incubation with probe **5** or the appropriate controls (DMSO or Biotin-Ub) at 37 °C for 2 h (Figure 3A).

When comparing the proteins enriched by probe **5** between the infected and noninfected sample groups, both DupA and DupB were significantly enriched, confirming the potential to apply the guanidinium-linked probe in a complex *Legionella* effector-containing proteome (Figure 3B). In analogy to the triazole-linked probe, the guanidinium probe only significantly targets DupA and DupB within the *Legionella* proteome.^15^ When comparing probe **5** to the biotin-Ub control within the *Legionella*-infected cells, DupA and DupB also showed significant enrichment (Figure 3C), whereas when comparing biotinylated Ub versus DMSO, the Dups fall below the significance threshold (Supplementary Figure 4). This underscores the warhead to be crucial in effectively pulling down the Dups, and noncovalent interactions are insufficient in the used protocol. When considering the mammalian proteins captured by probe **5**, differences between infected cells and noninfected cells were observed (Figure 3B). Interestingly, mammalian proteins associated with the DNA damage response^16,17^ including PARP1, XRCC5, and XRCC6 are enriched and depicted in the left panel, indicating a substantial decrease of protein expression or reactivity toward the probes during *Legionella* infection. Upon further analysis of the enriched mammalian proteins, the identified proteins are categorized into different clusters, similarly to our recent study,^12^ based on their respective roles in regulating Ub dynamics (green), previously reported SidE-targets (purple),^3,7^ potential Cys-reactivity toward the warhead^18,19^ (pink), and recognition of structural properties of the ribosyl moiety (orange) (Supplementary Figure 4). Analogous to the triazole-linked probe, the guanidinium derivative captured a significant number of Ub-regulatory proteins. These include Ub-conjugating enzymes (NEDD4L, ARIH1, RNF25, and TRIM65), Ub proteases (USP3, USP14, USP19, USP48, OTUB1, and USP15), and Ub receptors (ADRM1, PMSD4) (Figure 3B,C). Notably, E1 enzymes for both Ub (UBA1) and other Ub-like proteins—Nedd8 (UBA3), SUMO (SAE2), and LC3 (ATG7)—were also captured. These E1 enzymes share the initial AMPylation activity on the C-terminus of their target Ub-like proteins, suggesting that the structural similarity of probe **5** to an AMP-activated Ub(l) might trigger E1 reactivity. Several proteins previously identified as PR-ubiquitination targets, including the GTPases RAB14, RAB5C, RAB21, and the ER-associated protein RTN4, were also enriched (Supplementary Figure 4).

A *t-*test comparison of both triazole-linked and guanidinium-linked probes in *Legionella*-infected samples against biotin-Ub further showcases the general overlap in cysteine reactivity (toward, e.g., PDIAs), riboside-associated recognition, Ub-binding, and PR-target enrichment (Figure 3C). Notably, while comparing the enrichment by both probes between the *Legionella*-infected samples and the noninfected control samples, both Dups were observed. Additionally, a negative effect on the enrichment of DNA damage response proteins PARP1, XRCC5, and XRCC6 in both probe sets stands out.

Altogether the guanidinium probe is able to enrich DupA and DupB as the most significant proteins. Differences, but also overlapping enriched proteins identified between the guanidinium- and triazole-linked probes, are observed. Subtle differences in the chemical nature or orientation of the sugar moiety and attached warhead, due to the different linkages, might account for these observations. In addition, the guanidinium-linked probes carry the native occurring connection that might also be broken down by glycohydrolases in the cellular environment, resulting in a loss of active probe during the incubation prior to the pull-down protocol.

## Conclusions

To conclude, we report on the development of a methodology to synthesize native arginine-linked Ub-phosphoribose mimics that act as covalent probes to target *Legionella* effector enzymes. The strategy to synthesize these probes via equipping isothiourea ribosides with the fluoro-sulfonate or vinyl-sulfonate warheads prior to on-resin coupling on the free amine in Ub_1–76_ (Arg42Orn) led to the successful production of natively guanidinium-linked fluoro-sulfonate and vinyl-sulfonate probes **1** and **2**. The reactivity of both probes on recombinant *Legionella* hydrolase DupA as well as *Legionella* ligase SdeA_PDE_ was evaluated, and the formation of the covalent complex was observed in all cases, although with reduced conversion efficiency when compared to the triazole-linked probe analogues. Both DupA and DupB were successfully enriched from *Legionella*-infected HEK293T lysate in a pull-down proteomics experiment using the native arginine-linked probe **5**. Of note, the SidE effectors were not enriched in this pull-down experiment, although binding to the probe on recombinant SdeA_PDE_ was verified. This is potentially due to the fact that the *Legionella*-infected cells in this study were lysed 4 h postinfection, and previous research indicates that the activity of SdeA is concentrated in the initial period of infection and is significantly reduced 4 h post infection.^20^ Overall, our experiments confirmed the applicability of the natively linked probe to a complex proteome and demonstrate that concerns related to enzymatic processing^21,22^ of the guanidium bond or the probe being an anomeric mixture are no major reasons to avoid the application of such probes.^10^ The guanidinium Ub-phosphoribose probes effectively bind *Legionella* enzymes at the recombinant protein level and in *Legionella*-infected lysate and therefore complement the PR-ubiquitination toolbox to study *Legionella* effectors. Our previous study and the current work show the cross-linking capacity of vinyl-sulfonate-PR-Ub probes toward Cys196 of DupA but also to Lys197, albeit with slower kinetics.^12^ We believe that the future utilization of these probes could aid in detecting Dups and SidE ligases at various stages of infection, potentially providing valuable insights into the presence of these effectors during different phases of the infection process. Furthermore, these probes may enable competitive screening for covalent inhibitors, acting on Dup Cys196, potentially blocking access to the enzyme’s active site. Additionally, as the developed probes target the PR-ubiquitination pathway utilized by *Legionella pneumophila*, these probes could help identify the yet unidentified homologous effectors used by other pathogenic bacteria. This could reveal whether the PR-ubiquitination mechanism represents a broader strategy employed by diverse pathogens to hijack host ubiquitination systems.

## Data Availability

The mass spectrometry proteomics data have been deposited to the ProteomeXchange Consortium via the PRIDE partner repository with the data set identifier *PXD049797*.

## Supporting Information Available

Experimental procedures, NMR-analysis, mass spectra, synthetic details, annotated MS/MS spectra, and proteomic assessment (PDF)

## List of Abbreviations

AMP: adenosine monophosphate
ADPr: adenosine diphosphate ribosyl
PR: phosphoribosyl
PR-ubiquitination: phosphoribosyl serine ubiquitination
DUP: deubiquitinase for PR-ubiquitination
LCV: *Legionella*-containing vacuole
mART: monoADPribosyl transferase
PDE: phosphodiesterase
CuAAC: copper-catalyzed azide–alkyne cycloadditions
WT: wild-type
HPLC: high-performance liquid chromatography
TBDPS: *tert*-butyldiphenylsilyl
TBAF: *tetra-n-butylammoniumfluoride*
SDS-PAGE: sodium dodecyl-sulfate polyacrylamide gel electrophoresis
