# Supplementary material for: Comparison of Phosphoribosyl Ubiquitin Probes Targeting Legionella Dup Enzymes
Source: Bioconjug Chem. 2025 Feb 17;36(3):457–63. doi: 10.1021/acs.bioconjchem.4c00541 (PMC11926780; doi:10.1021/acs.bioconjchem.4c00541)
Supplement: Supplementary file 1 — bc4c00541_si_001.pdf [file bc4c00541_si_001.pdf]

Comparison of phospho-ribosyl Ubiquitin probes targeting Legionella Dup enzymes

Max S. Kloet[a], Rishov Mukhopadhyay[a], Rukmini Mukherjee[b], Mohit Misra[b], Cami M. P. Talavera Ormeño[a], Rayman T. N. Tjokrodirijo[c], Paul J. Hensbergen[c], Peter A. van Veelen[c], Ivan Đikić[b], Aysegul Sapmaz[a], and Gerbrand J. van der Heden van Noort[a]*

a Department of Cell and Chemical Biology, Leiden University Medical Centre, Leiden, The Netherlands

b Buchmann Institute for Molecular Life Sciences, Goethe University, Frankfurt, Germany

c Center for Proteomics and Metabolomics, Leiden University Medical Center, Leiden, The Netherlands

Supporting Information

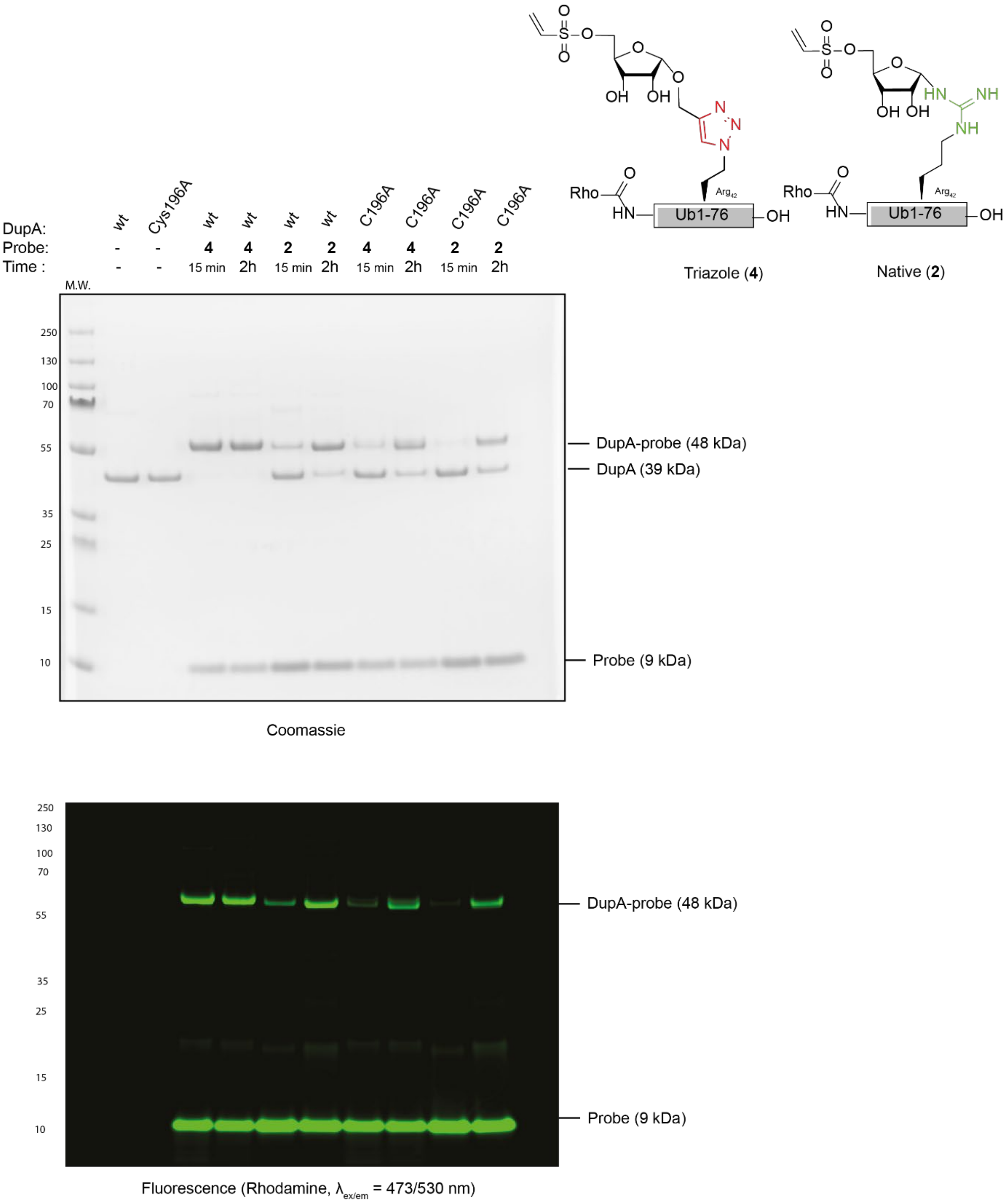

Supplementary Figure 1. Natively or triazole linked vinyl-sulfonate probes **2** and **4** show differences in labeling towards DupA WT and DupA Cys196Ala. SDS-PAGE analysis of the reaction between recombinant DupA Wt or the Cys196Ala mutant and vinyl-sulfonate probes **2** or **4**. DupA was subjected to 8 equivalents of probe **2** or **4** and incubated at 37 °C for the indicated time points. The upper panel is Coomassie stained and the bottom panel represents a rhodamine fluorescence scan ($\lambda_{ex/em}$ = 473/530 nm).

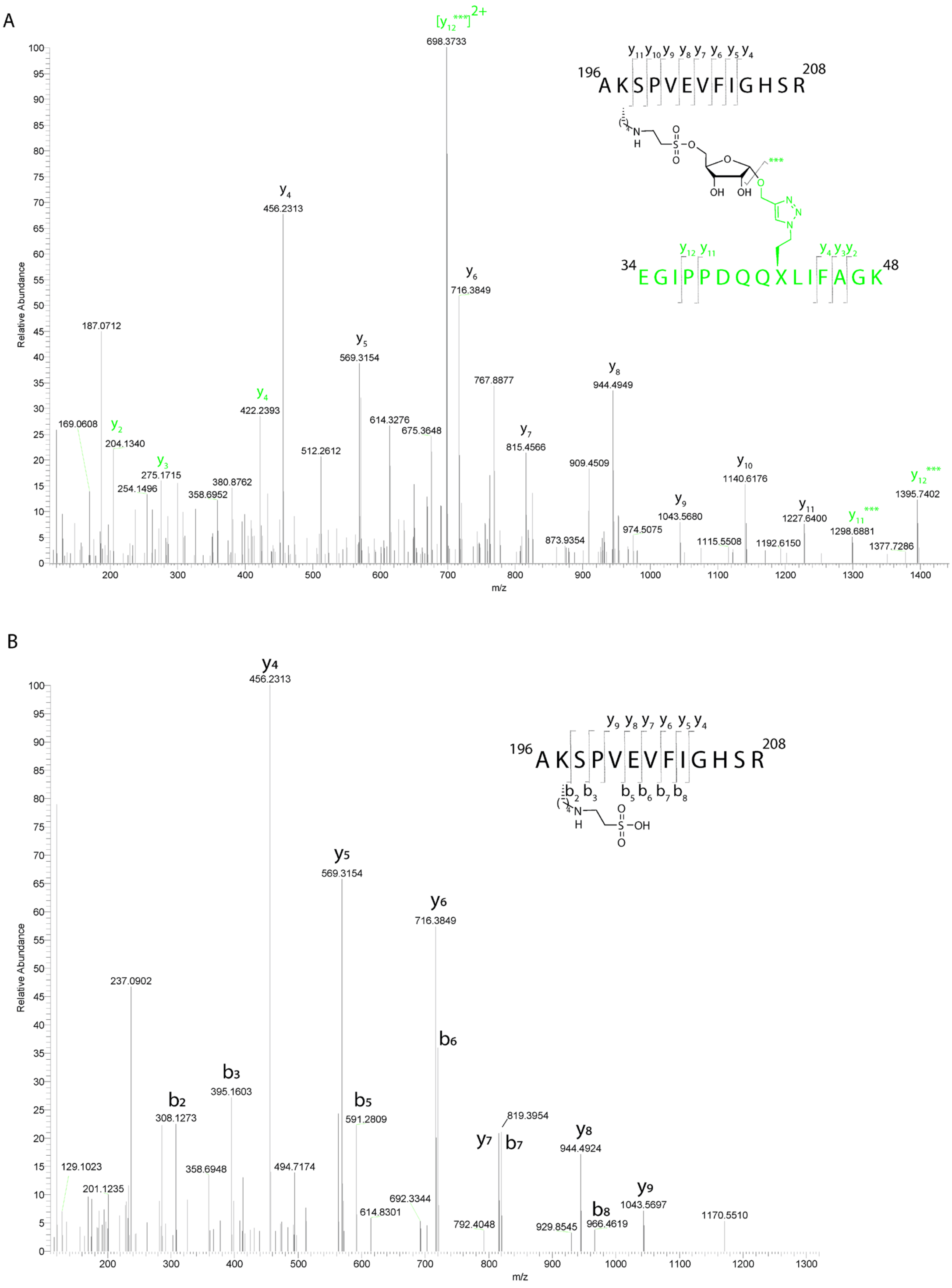

Supplementary Figure 2. Annotated MS/MS spectra of the crosslinking-peptide from DupA Cys196Ala mutant:probe **4** complex. **A)** The MS/MS spectrum of tryptic peptide of Ub[34-48] crosslinked to DupA [196-208]. The y-ion series of the DupA Cys196Ala peptide are in black and the y-ion series of the Ub peptide in green.*** indicates fragmentation site at anomeric position of ribose. **B)** Tryptic peptide of DupA [196-208] carrying only part of the linker to Ub probe **4**, probably due to hydrolysis in sample preparation. The b-ion series identifies the crosslinking remnant to be attached to Lys^{197} of DupA. The missed cleavage of trypsin after Lys in both peptides from **A** and **B** further indicate that the Lys residue is the site of crosslinking towards Ub probe **4**.

A

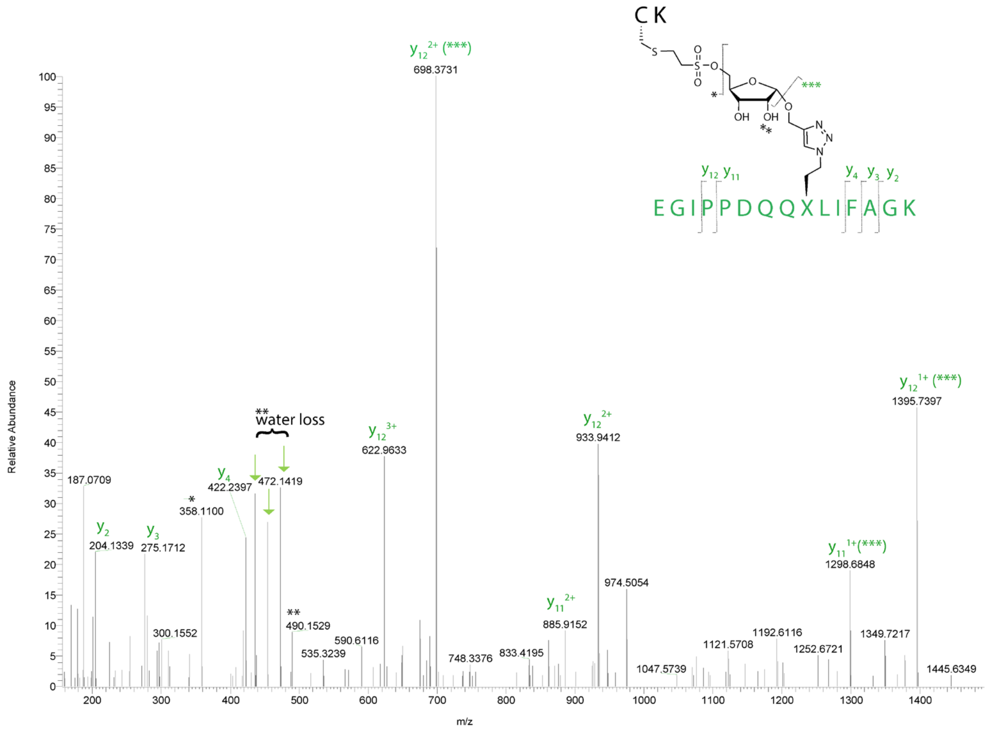

B

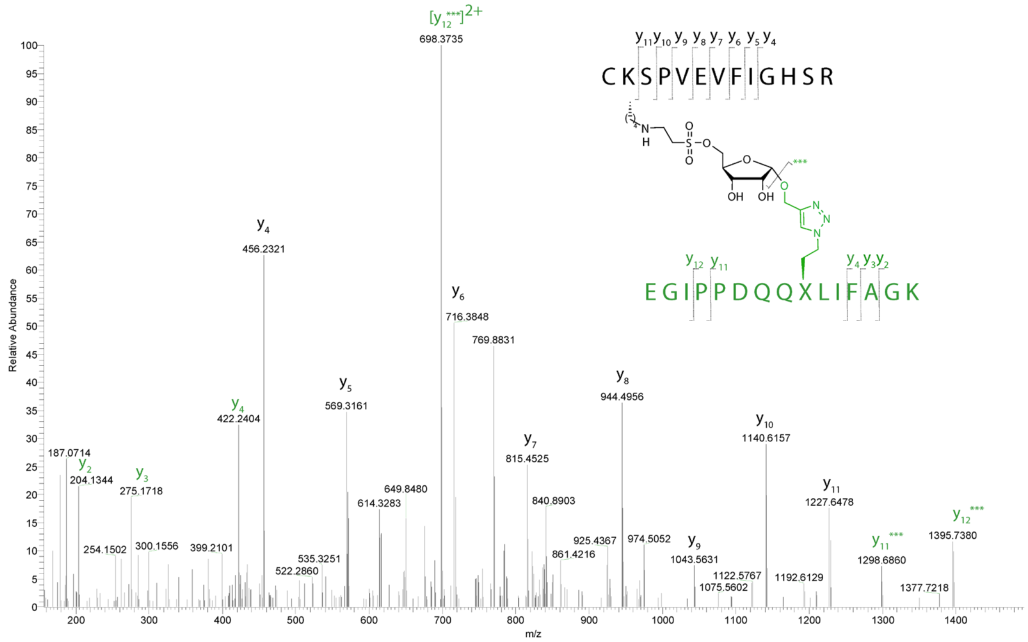

Supplementary Figure 3. Annotated MS/MS spectra of the crosslinked peptides from the DupA WT:probe **4** complex. **A)** The MS/MS spectrum of the tryptic peptide of Ub[34-48] crosslinked to DupA [196-197]. * indicates fragmentation after sulfonate linker, ** and *** indicate fragmentation at anomeric position of ribose. The tryptic peptide Cys^{196}Lys^{197}, indicates the crosslinking to be at Cys^{196} as normal trypsin activity (cleaving after unmodified Lys) is observed, **B)** The missed cleavage after Lys resulting in the longer DupA [196-208] tryptic peptide crosslinked to Ub[34-48] indicates the crosslinking to be on Lys^{197}, which is further substantiated by the corresponding b-ions of the analogues Cys196Ala peptide in Sup. Fig. 2B.

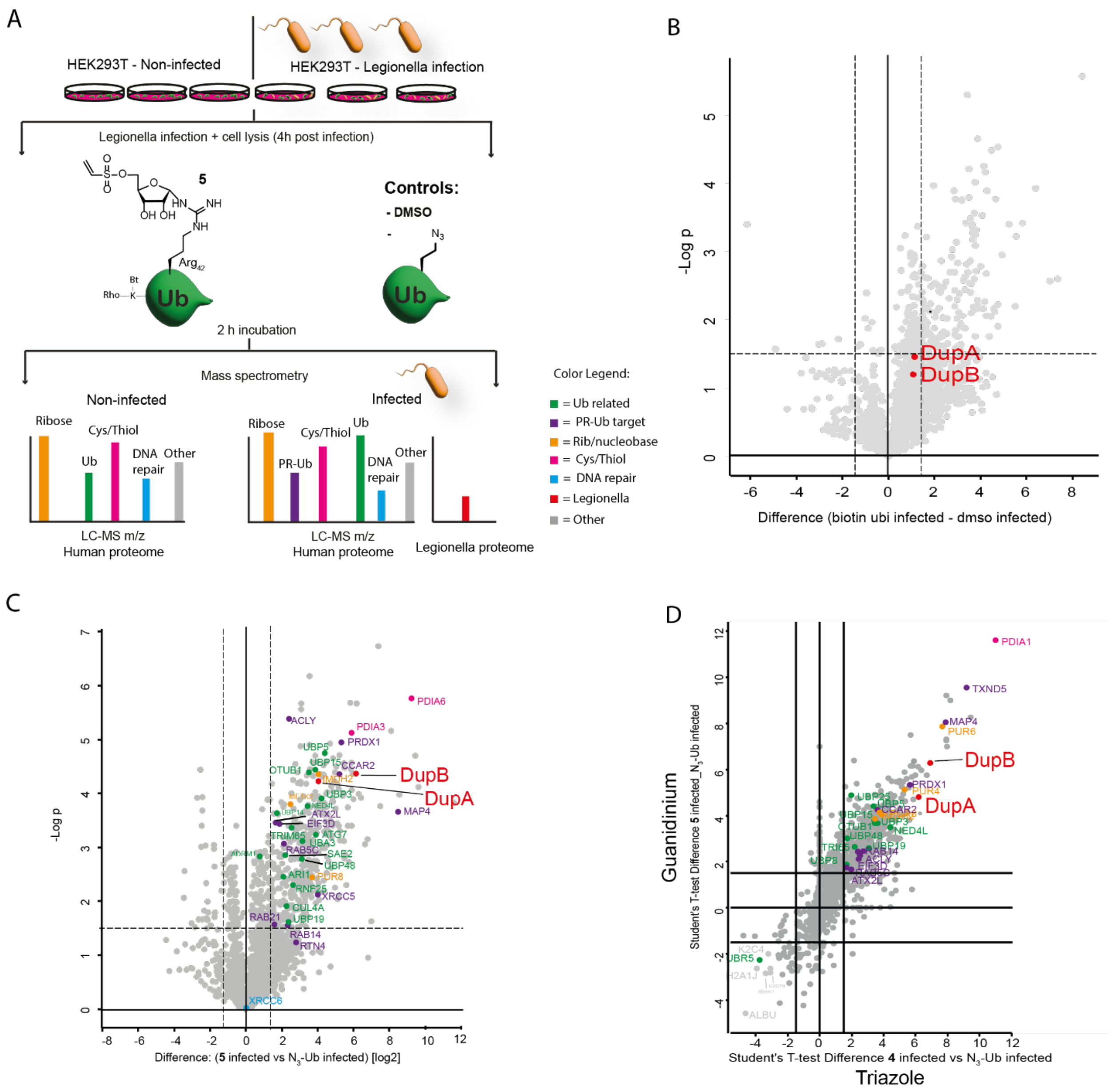

Supplementary Figure 4. Proteomic assessment of the pulldown by the guanidinium linked vinyl-sulfonate probe **5** from non-infected HEK293T cells or infected with Legionella, **A)** Schematic representation of the workflow applied in mass spectrometry-based proteomics. The lysates of two samples groups; infected with Legionella and non-infected, were prepared in triplicate. **B)** volcano plot comparing biotin-Ub and DMSO in the legionella infected sample group, highlighting DupA and DupB not being significantly enriched. **C)** Volcano plot showing the significant enriched proteins by probe **5** compared to biotin-Ub within the infected sample group. **D)** Student's T-test analysis comparing enrichment by probe **5** normalized to biotin-Ub, to triazole probe **4** normalized for biotin-Ub, in the Legionella-infected sample group. For the plot holds: black dashed lines correspond to the thresholds: log_2 ratio ≥1.5; p-value ≤0.05. A color code legend is provided for the clustered proteins. The Legionella enzymes DupA and DupB are highlighted in red.

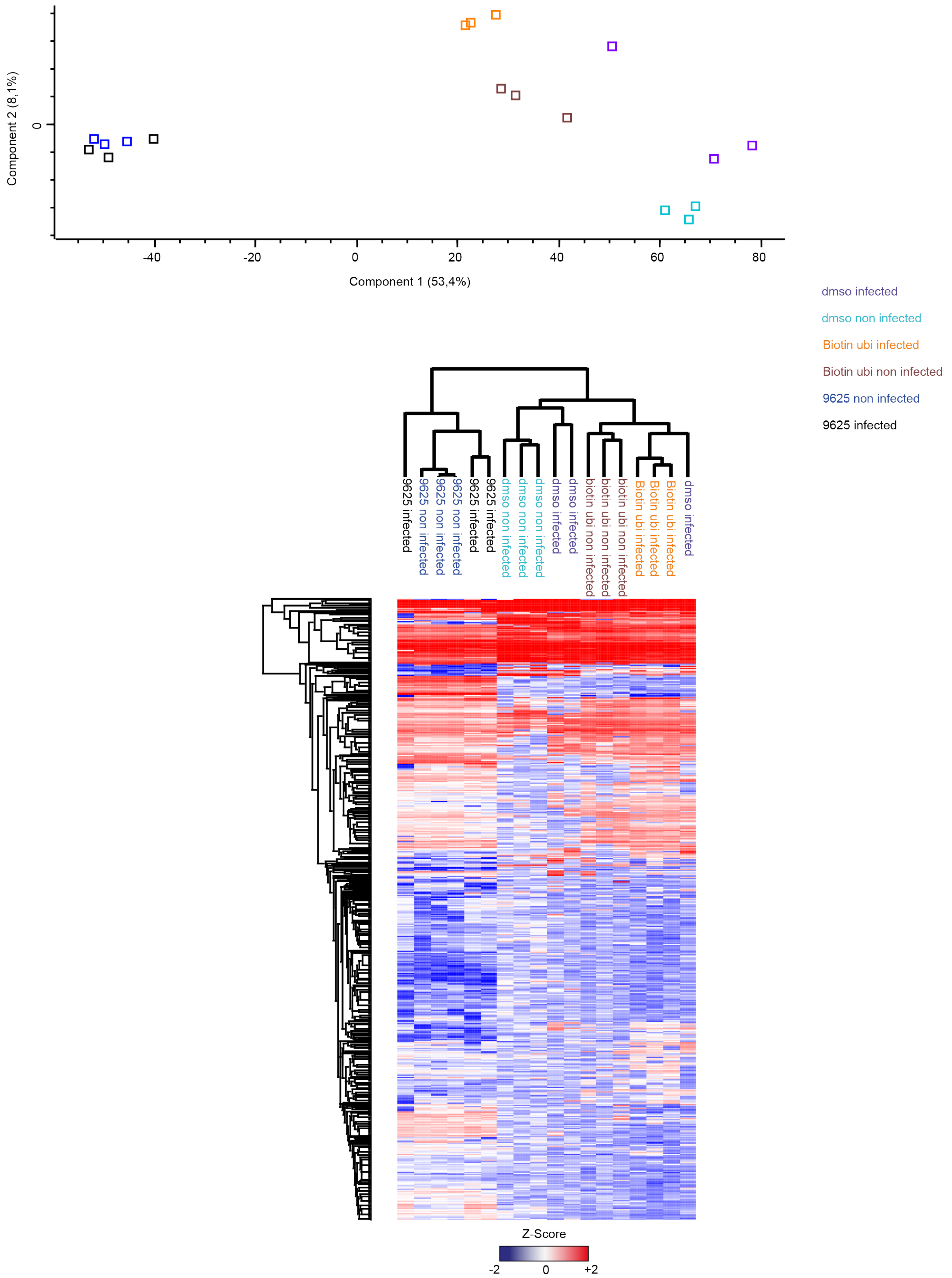

Supplementary Figure 5 : Principal component analysis of the DIA data was done using components with the highest explained variance, n = 3. Heat map shows Hierarchical clustering of the significantly affected genes (p value < 0.05) from the proteome data set. Clustering done via Euclidean distance (pre-processed with k-means, 300 clusters, 1000 iterations), where each column in the heatmap is a sample and each row is a protein. The LFQ intensity values were normalized by Z-score. Probe **5** is annotated as ‘9625’.

'*In-Eppendorf-o*' labeling assay of probes 1-4 to recombinant DupA Wt, DupA active site mutants and SdeA PDE

The probes **1-4** (27.4 µM, 8.13 eq.) in buffer (20 mM TRIS, 150 mM NaCl, pH 7.6) were placed in eppendorf tubes and incubated with DupA Wt, a DupA active site mutant or SdeA PDE (3.4 µM) at 37 °C in a total volume of 26.66 µL. After incubation at the indicates time points (15 min, 2 h or 4 h, **Fig 1.**), 10 µL sample was taken and added to 5 µL loading buffer including β-mercaptoethanol. Samples were run on a NuPAGE™ 4-12% Bis-Tris gel in MES buffer at 190 mV for 45 minutes. A fluorescence scan on a Typhoon FLA 9500 (rhodamine channel, $\lambda_{ex/em}$ = 473/530 nm) was performed to visualize the complex formed and additionally, the proteins were stained with Coomassie staining. SDS PAGE analysis is depicted in **Fig 1**.

Chemoproteomics in Legionella-infected HEK293T cells

Cell lines

HEK293T expressing CD32 were cultured in DMEM supplemented with 10% FBS, 100 I.U./mL penicillin and 100 mg/mL streptomycin (Pen/Strep) at 37 °C, 5% CO_2.

Legionella pneumophila culture and infection

Wild type L. pneumophila (Lp02) were grown for 3 days on N-(2-acetamido)-2-amino-ethanesulfonic acid (ACES)-buffered charcoal-yeast (BCYE) extract agar, at 37 °C, followed by growth for 20 h in ACES yeast extract media. Bacterial cultures of optical density between 3.2-3.6 were used to infect cells at an MOI of 1:10.

Preparation of cell lysate from Legionella infected cells.

Legionella infected or non-infected HEK293T cells growing on a 10 cm dish were lysed in KHEM lysis buffer (20 mM Hepes-KOH (pH =7.5), 150 mM KCl, 2 mM $MgCl_2$, 0.2 mM EDTA, 1% Triton-X100, protease inhibitor cocktail) 4 hours post infection. For lysis, cells were collected in PBS, centrifuged at 800 rcf to get a cell pellet. Lysis buffer was added to the cell pellet and incubated in ice for 10 min. This was then centrifuged at 15000g for 10 min. The pellet was discarded and the supernatant was used as the cell lysate for the pull-down assay.

Pull-down assay with biotin conjugated chemical probes

For each sample, 300 µl of lysate was incubated with 2 µM of one of the following conditions: 1) DMSO control, 0.36 µL. 2) Biotin-ubiquitin (Arg42 → Azido homoalanine) (0.3 µL of a 2 mM solution in DMSO, 2 µM final concentration) 3) Vinyl-sulfonate probe **5** (0.3 µL, 20 µg/µL, 2 mM in DMSO). Lysates were Incubated with the probes and controls at 37 °C for 2 hours. Streptavidin-agarose resin was equilibrated in lysis buffer. 30 µL of equilibrated resin was added to the lysate-probe mixture and the total volume of the reaction was adjusted to 1 mL by adding 700 µL of lysis buffer. This was then incubated overnight at 4 °C on a rotator. On the next day, the resin was washed with wash buffer (Tris 20 mM, NaCl 150 mM, pH 7.5, 2% SDS) by centrifuging at 500 rcf for 1min followed by removal of the supernatant. The wash was repeated 6 times; the resin was then transferred to new microfuge tubes and boiled in SDS with β-mercaptoethanol for 15 min. Subsequently, the samples were run on SDS-PAGE followed by Coomassie Blue staining. This was then subjected to in-gel trypsin digestion and mass-spectrometry. The experiment was performed in biological triplicates for both non-infected and Legionella infected HEK293T cells.

Proteomics mass spectrometry measurements

Samples were loaded onto a 4-12 % Bis-Tris gradient gel (Invitrogen) and run for 1.5 cm. Subsequently, each lane was cut into four bands. Gel slices were first washed 3x with water, and subsequently subjected to reduction with 10 mM dithiothreitol, alkylation with 50 mM of iodoacetamide, and in-gel trypsin digestion using a Proteineer DP digestion robot (Bruker). After addition of trypsin (at 12.5 ng/µL) and swelling of the bands, the bands were transferred to Eppendorf vials and the bands were covered in 25 mM NH_4HCO_3 pH 8.3. Tryptic digestion took place overnight at 37 °C and the peptides were extracted from the gel slices with with 50/50/0.1 v/v/v water/acetonitril/formic acid. Finally peptides were lyophilized. The experiment was performed in biological triplicates for both non-infected and Legionella infected HEK293T cells.

Tryptic peptides were dissolved in water/formic acid (100/0.1 v/v) and subsequently analyzed by on-line C18 nanoHPLC MS/MS with a system consisting of an Ultimate3000nano gradient HPLC system (Thermo, Bremen, Germany), and an Exploris480 mass spectrometer (Thermo). Fractions were injected onto a cartridge precolumn (300 µm × 5 mm, C18 PepMap, 5 um, 100 A, and eluted via a homemade analytical nano-HPLC column (50 cm × 75 µm; Reprosil-Pur C18-AQ 1.9 um, 120 A (Dr. Maisch, Ammerbuch, Germany). The gradient was run from 2% to 40 % solvent B (20/80/0.1

water/acetonitrile/formic acid (FA) v/v) in 30 min. The nano-HPLC column was drawn to a tip of ~10 µm and acted as the electrospray needle of the MS source. The mass spectrometer was operated in data-dependent MS/MS mode for a cycle time of 3 seconds, with a HCD collision energy at 30 V and recording of the MS2 spectrum in the orbitrap, with a quadrupole isolation width of 1.2 Da. In the master scan (MS1) the resolution was 120,000, the scan range 400-1500, at standard AGC target @maximum fill time of 50 ms. A lock mass correction on the background ion m/z = 445.12 Da was used. Precursors were dynamically excluded after n=1 with an exclusion duration of 10 s, and with a precursor range of 20 ppm. Charge states 2-5 were included. For MS2 the first mass was set to 110 Da, and the MS2 scan resolution was 30,000 at an AGC target of 100% at maximum fill time of 60 ms.

In a post-analysis process, raw data were first converted to peak lists using Proteome Discoverer version 2.2 (Thermo Electron), and submitted to the combined Uniprot database (Homo sapiens, 20596 entries, and the Legionella pneumophila subsp. pneumophila (strain Philadelphia 1 / ATCC 33152 / DSM 7513), UP000000609, 2930 entries), using Mascot v. 2.2.07 (www.matrixscience.com) for protein identification. Mascot searches were with 10 ppm and 0.02 Da deviation for precursor and fragment mass, respectively, and trypsin as enzyme. Up to two missed cleavages were allowed. Methionine oxidation and acetyl on protein N-terminus were set as a variable modification; carbamidomethyl on Cys, were set as a fixed modification. Protein FDR was set to 1 %. Normalization was on total peptide amount.

Bioinformatic analysis of DIA data:

Peptide intensity table with the Label free quantitation (LFQ) values were analyzed in Perseus (v1.6.2.3). Data were log2 transformed and filtered for identification in all three replicates in at least one group. Principal component analysis (PCA) was performed for each analysis with default settings. Intensities were *Z* scored by subtracting the mean and used for hierarchical clustering by Euclidean distance (pre-processed with k-means, 300 clusters, 1000 iterations) (**Supplementary Fig. 3**). Missing values were imputed from the lower end of the normal distribution (default settings). A two-sided student's *t* test with permutation-based FDR was used to calculate significance between probe pulldown and control with/without infection, at 0.05 FDR (*p* value).

Chemical synthesis

General synthetic procedures

All reagents were used as received unless stated otherwise. Solvents used in synthesis were dried and stored over 4Å molecular sieves, except for MeOH and MeCN which were stored over 3Å molecular sieves. Triethylamine (TEA) and diisopropylethylamine (DIPEA) were stored over KOH pellets. Column chromatography was performed on silica gel 60 Å (40-63 µm, Macherey-Nagel). TLC analysis was performed on Macherey-Nagel aluminium sheets (silica gel 60 F_{254}). TLC was used to visualize compounds by UV at wavelength 254 nm and by spraying with either cerium molybdate spray (25 g/L $(NH_4)_6Mo_7O_{24}$, 10 g/L $(NH_4)_4Ce(SO_4)_4·H_2O$ in 10% H_2SO_4 water solution) or $KMnO_4$ spray (20 g/L $KMnO_4$ and 10 g/L K_2CO_3 in water) followed by charring at c.a. 250 °C. NMR spectra were recorded on a Bruker AV-300 NMR. Chemical shifts (δ) are given in ppm relative to tetramethyl silane. Coupling constants (*J*) are given in Hz. All given ^{13}C-APT spectra are proton decoupled.

LC-MS measurements and HPLC purifications

LC-MS measurements were performed on a Waters ACQUITY UPLC system equipped with a Waters ACQUITY Quaternary Solvent Manager (QSM), Waters ACQUITY UPLC Photodiode Array (PDA) eλ Detector (λ = 210-800 nm) and Waters ACQUITY UPLC Protein BEH C18 column (1.7 µm, 2.1 x 50 mm) and LCT Premier Orthogonal Acceleration Time of Flight Mass Spectrometer (m/z = 100-1600) in ES+ mode. Samples were run using 2 mobile phases: A = 100% C_3CN, 0.1% formic acid in water and B = 1% water and 0.1% formic acid in CH3CN. Flow rate= 0.5 mL/min, runtime= 3 min, column T= 40°C. Gradient: 0 - 95% B. Data processing was performed using Waters MassLynx Mass Spectrometry Software 4.1 (deconvolution with MaxEnt1 function).

HPLC purification was performed on a **A)** Shimadzu semi-preparative RP-HPLC system, equipped with a Waters C18-Xbridge 5 µm OBD (10 x 150 mm) column at a flowrate of 6.5 mL/min. using 2 mobile phases: A: MQ + 0.05% FA, B: MeCN + 0.05 % FA. Gradient: 10 -> 70% B. HPLC system **B**) Waters preparative RP-HPLC system, equipped with a Waters C18-Xbridge 5 µm OBD (30 x 150 mm) column at a flowrate of 37.5 mL/min using 3 mobile phases: A: MQ, B: CH3CN and C: 1% TFA in MQ. Gradient: 20 -> 45% B, 5% C. High resolution mass spectra were recorded on a Waters XEVO-G2 XS Q-TOF mass spectrometer equipped with an electrospray ion source in positive mode

(source voltage 3.0 kV, desolvation gas flow 900 L/hr, temperature 250 °C) with resolution R = 22000 (mass range m/z = 50-2000) and 200 pg/uL Leu-Enk (m/z = 556.2771) as a "lock mass".

Solid Phase Peptide Synthesis (SPPS)

SPPS was performed according to literature procedure[22] on a Syro II MultiSyntech Automated Peptide synthesizer using standard 9-fluorenylmethoxycarbonyl (Fmoc) based solid phase peptide chemistry at 20 µmol scale, using fourfold excess of amino acids relative to preloaded Fmoc-Gly wang resin (0.2 mmol/g, Rapp Polymere GmbH). On position-42 in the peptide sequence arginine was replaced by Fmoc-Orn(Alloc)-OH.

1-(*tert*-butoxycarbonyl)-2,3-di-*O*-(4-methoxybenzyl)-α-D-ribofuranos-1-yl)-2-ethylisothiourea (7)

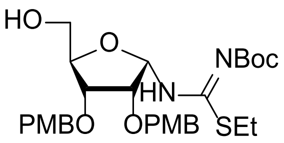

α-linked anomeric isothiourea N-riboside **6** (113 mg, 0.138 mmol) as described by Kloet *et al.,*[13] was dissolved in THF (0.66 mL) before TBAF (145 µL, 1M in THF, 0.145 mmol, 1.1 eq.) was added. After stirring for 2 hours at rt the reaction mixture was concentrated *in vacuo*. The residue was purified by silica column chromatography (0 → 60% EtOAc in Heptane) to obtain **7** (56 mg, 0.097 mmol, 71%) as a colorless oil. ^{1}H NMR (300 MHz, $CDCl_3$) δ 7.33 – 7.27 (m, 2H), 7.27 – 7.20 (m, 2H), 6.89 – 6.83 (m, 4H), 5.56 (d, *J* = 3.3 Hz, 1H), 4.62 (d, *J* = 2.3 Hz, 2H), 4.47 (dd, J = 12, 9 Hz, 2H), 4.16 – 4.08 (m, 2H), 4.03 (t, *J* = 5.5 Hz, 1H), 3.85 (dd, *J* = 5.1, 3.4 Hz, 1H), 3.80 (s, 6H), 3.56 (dd, *J* = 12.1, 2.7 Hz, 1H), 3.07 (q, *J* = 7.4 Hz, 2H), 1.50 (s, 9H), 1.32-1.28 (m, 3H). ^{13}C NMR (75 MHz, $CDCl_3$) δ 159.6, 159.5, 129.9, 129.8, 129.7, 129.5, 114.0, 87.0, 82.4, 80.2, 79.8, 75.8, 72.2, 72.0, 61.7, 55.4, 28.3, 25.5, 13.9.

1-(*tert*-butoxycarbonyl)-3-(5-*O*-((*Fluoro-sulfonate*)-2,3-di-*O*-(4-methoxybenzyl)-α-D-ribofuranos-1-yl)-2-ethylisothiourea (8)

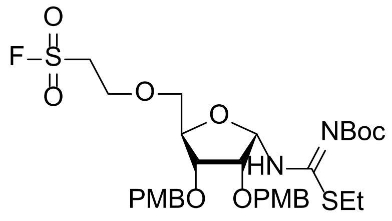

Isothiourea Riboside **7** (60 mg, 0.26 mmol) was co-evaporated with anhydrous MeCN and subsequently placed under argon atmosphere and dissolved in anhydrous DCM (651 µL). Et_3N (54 µL, 0.39 mmol, 3 eq.) and ethenesulfonyl fluoride (19.8 µL, 0.26 mmol, 2 eq.) were added and the reaction mixture was stirred at rt overnight. The reaction mixture was subsequently concentrated *in vacuo* and the residue purified by silica column chromatography (0 → 70% EtOAc in Heptane) to obtain **8** (54 mg, 0.079 mmol, 61%) as a colorless oil. ^{1}H NMR (300 MHz, Chloroform-*d*) δ 7.31 (dd, *J* = 8.9, 2.5 Hz, 3H), 7.26 – 7.21 (m, 2H), 6.90 – 6.83 (m, 5H), 5.56 (s, 1H), 4.61 (d, *J* = 4.0 Hz, 2H), 4.45 (d, *J* = 23.0 Hz, 2H), 4.22 – 4.04 (m, 1H), 4.16 – 4.03 (m, 1H), 3.99 – 3.88 (m, 2H), 3.80 (s, 6H), 3.69 – 3.47 (m, 4H), 3.11 (qd, *J* = 7.4, 4.0 Hz, 2H), 1.49 (s, 9H), 1.30 (t, *J* = 7.4 Hz, 3H). ^{13}C NMR (75 MHz, Chloroform-*d*) δ 161.6, 159.5, 129.9, 129.8, 129.7, 129.6, 113.9, 87.5, 81.1, 80.7, 79.5, 76.4, 72.3, 72.0, 70.2, 64.4, 55.4, 50.8, 50.6, 28.3, 25.5, 14.0. ^{19}F NMR (282 MHz, Chloroform-*d*) δ 58.48.

Rho-Ub$_{1-76}$(Arg42 → native arginine linked fluoro-sulfonate riboside) (1)

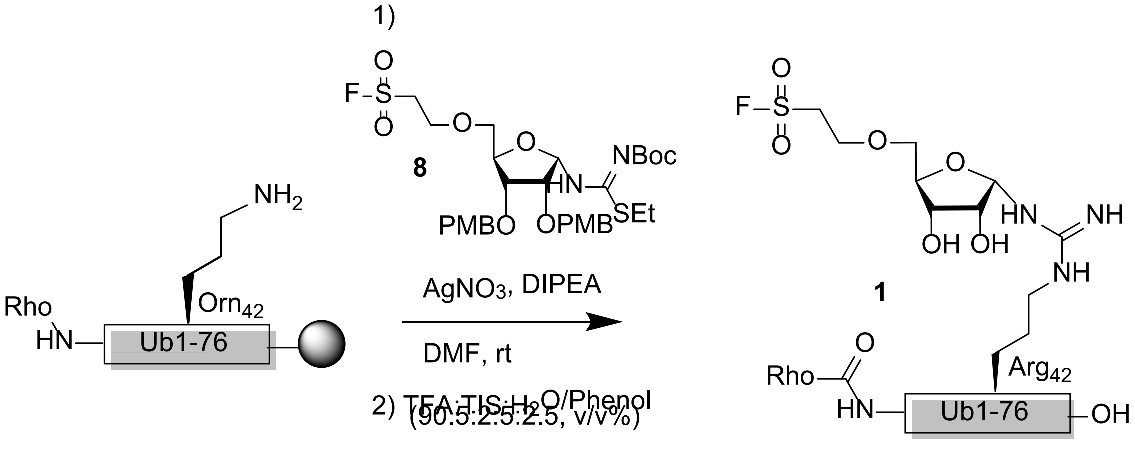

10 µmol Ub$_{1-76}$ (Arg42Orn(Alloc)) on resin was treated with PyBOP (26 mg, 50 µmol) and DiBoc-Rhodamine (28.7 mg, 50 µmol, 5 eq.) in DMF (2 mL). After 5 min of shaking, DIPEA (26 µL, 150 µmol, 15 eq.) was added. The reaction mixture was shaken for 1.5 hours, after which a test cleavage confirmed full conversion into rhodamine conjugate (mass found: (M + H^+ = 8945). Next, the resin was washed with DMF followed by DCM. Alloc deprotection was performed subsequently by the addition of ($Pd(PPH_3)_4$ (2.3 mg, 2 µmol, 0.2 eq.) and PhSiH (24.6 µl, 200 µmol, 20 eq.) in DCM (2 mL). After shaking for 15 min, the resin was washed with DMF and DCM before repeating the Alloc deprotection procedure twice. A test cleavage confirmed removal of the Alloc group in **10** (mass found: (M + H^+ = 8861).

*$AgNO_3$ Mediated coupling of resin **10** to compound **8***

1 µmol Rho-Ub$_{1-76}$ (Arg42Orn) **10** was swelled in DMF before treated with a solution of fluoro-sulfonate isothiourea N-riboside **8** (10.35 mg, 15 µmol, 15 eq.) in DMF (150 µL, 0.05M). Subsequently, TEA (21 µL, 150 µmol, 150 eq.) and were added $AgNO_3$ (2.54 mg, 15 µmol, 15 eq.) followed by another 100 µL of DMF. The syringe was subsequently wrapped in aluminum foil to protect it from light. The resin was shaken for 4 hours before a test cleavage confirmed formation of the conjugate (M + H^+ = 9146). The resin was then extensively washed with DMF and DCM before the resin was treated with TFA/TIS/H_2O/Phenol (90.5/2/5/2.5, v/v) for 1 hour and 45 min before filtrated in an ice-cold solution of Et_2O:pentane (1:1, v/v). The precipitate formed was

centrifuged (5min, 3500 rpm) and the supernatant decanted. The pellet was subsequently dried with N_2, taken up in warm DMSO (1 mL) and diluted with warm water before purified by RP-HPLC. Pure fractions were pooled and lyophilized affording ubiquitin conjugate **1** (210 µg, 0.023 µmol, 2.3%) as a red powder. HRMS: $[C_{407}H_{656}FN_{107}O_{128}S + 7H]^{7+}$ found: 1307.5654, calculated:1307. 7714. $[C_{407}H_{656}FN_{107}O_{128}S + 8H]^{8+}$ found: 1144.2928, calculated: 1144.4250. $[C_{407}H_{656}FN_{107}O_{128}S + 9H]^{9+}$ found: 1017.2484, calculated: 1017.3777. $[C_{407}H_{656}FN_{107}O_{128}S + 10H]^{10+}$ found: 915.5858, calculated: 915.7400. $[C_{407}H_{656}FN_{107}O_{128}S + 11H]^{11+}$ found: 832.4414, calculated: 832.5818.

1-(*tert*-butoxycarbonyl)-3-(5-*O*-((*Vinyl-sulfonate*)-2,3-di-*O*-(4-methoxybenzyl)-α-D-ribofuranos-1-yl)-2-ethylisothiourea (9)

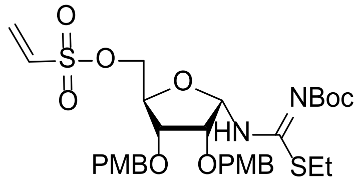

α-linked anomeric isothiourea N-riboside **7** (56 mg, 0.097 mmol) and Et_3N (67.4 µL 0.48 mmol, 5 eq.) were placed under argon atmosphere before dissolved in anhydrous DCM (0.47 mL). This was added to a stirring ice-cooled solution of 2-chloroethanesulfonyl chloride (20 µL, 0.19 mmol, 2 eq.) in anhydrous DCM (950 µL). The reaction mixture was stirred for 2 hours while cooled by a water-ice bath. Next, Sat. aq. $NaHCO_3$ was added and the organic phase separated and washed with H_2O. The organic layer was dried over $MgSO_4$, filtrated and concentrated *in vacuo*. The crude product was purified by silica column chromatography (0 → 70% EtOAc in Heptane) to yield vinyl-sulfonate **9** (31 mg, 0.047 mmol, 48%) as a colorless oil. ^1H NMR (300 MHz, $CDCl_3$) δ 7.38 – 7.26 (m, 2H), 7.33 – 7.24 (m, 2H), 6.89 – 6.83 (m, 4H), 6.49 (dd, J = 18, 12 Hz, 1H), 6.39 (d, *J* = 16.6 Hz, 1H), 6.12 (d, *J* = 9.5 Hz, 1H), 5.64 (s, 1H), 4.70 (d, *J* = 11.1 Hz, 1H), 4.60 (dd, J = 24, 9 Hz, 2H), 4.48 (d, *J* = 11.1 Hz, 1H), 4.25 (q, *J* = 3.3 Hz, 1H), 4.12 (d, *J* = 3.3 Hz, 2H), 4.05 – 3.98 (m, 2H), 3.80 (s, 6H), 2.91 (s, 2H), 1.50 (s, 9H), 1.21 (t, *J* = 7.5 Hz, 3H). ^{13}C NMR (75 MHz, $CDCl_3$) δ 161.2, 159.7, 159.6, 132.3, 130.8, 130.0, 130.0, 129.5, 129.3, 114.0, 79.6, 79.4, 76.5, 73.4, 73.1, 69.2, 55.4, 28.3, 25.3, 13.8.

Rho-Ub$_{1-76}$(Arg42 → native arginine linked vinyl-sulfonate riboside) (2)

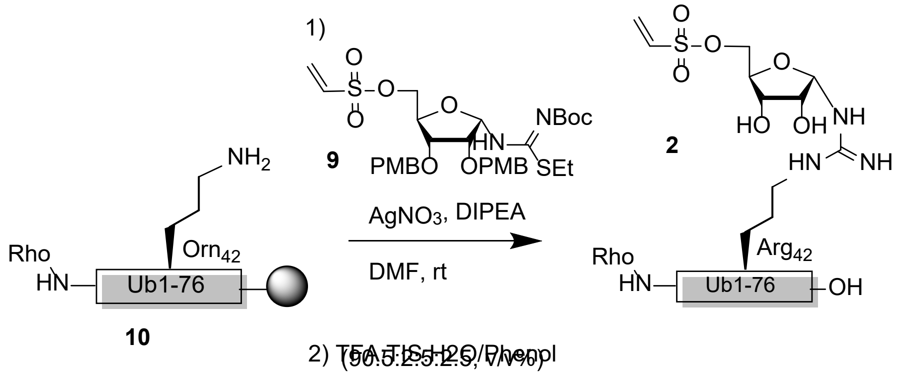

1 µmol Rho-Ub$_{1-76}$ (Arg42Orn) **10**, as prepared in the synthesis of **1**, was swelled in DMF before treated with a solution of vinyl-sulfonate isothiourea N-riboside **9** (10 mg, 15 µmol, 15 eq.) in DMF (150 µL, 0.05M). Et_3N (21 µL, 150 µmol, 150 eq.) and $AgNO_3$ (2.54 mg, 15 µmol, 15 eq.) were added followed by another 100 µL DMF. The syringe was subsequently wrapped in aluminum foil to protect it from light. The resin was shaken for 2.5 hours before a test cleavage confirmed formation of the conjugate (M + H^+ = 9125). The resin was then extensively washed with DMF and DCM before treated with TFA/TIS/H_2O/Phenol (90.5/2/5/2.5, v/v) for 1.5 hours and filtrated in an ice-cold solution of Et_2O:pentane (1:1, v/v). The precipitate formed was centrifuged (5min, 3500 rpm) and the supernatant decanted. The pellet was subsequently dried with N_2, taken up in warm DMSO (1 mL) and diluted with warm water before purified by RP-HPLC. Pure fractions were pooled and lyophilized affording ubiquitin conjugate **2** (560 µg, 0.061 µmol, 6.1%) as a red powder. HRMS: $[C_{407}H_{653}N_{107}O_{128}S + 7H]^{7+}$ found: 1304.5702, calculated: 1304.6257. $[C_{407}H_{653}N_{107}O_{128}S + 8H]^{8+}$ found: 1141.5103, calculated: 1141.6725. $[C_{407}H_{653}N_{107}O_{128}S + 9H]^{9+}$ found: 1014.8064, calculated: 1014.9311. $[C_{407}H_{653}N_{107}O_{128}S + 10H]^{10+}$ found: 913.3491, calculated: 913.5383. $[C_{407}H_{653}N_{107}O_{128}S + 11H]^{11+}$ found: 830.4518, calculated: 830.5811.

Rho-Lys(biotin)-PEG$_2$-Ub$_{1\text{-}76}$(Arg42 → native arginine linked vinyl-sulfonate ribose) (5)

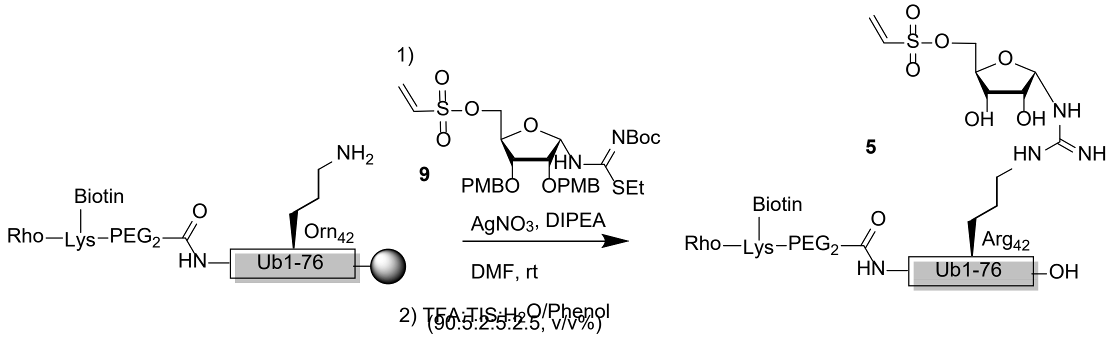

10 µmol Ub$_{1\text{-}76}$ (Arg42Orn(Alloc)) on resin was treated with HOBT (6.7 mg, 50 µmol, 5 eq), HBTU (19 mg, 50 µmol, 5 eq) and Fmoc-PEG$_2$-COOH (19.3 mg, 50 µmol, 5 eq) in DMF (2 mL). After 5 min of shaking DIPEA (26 µL, 150 µmol, 15 eq.) was added. The reaction mixture was shaken overnight, after which a test cleavage confirmed full conversion of the conjugation (mass found: (M + H$^+$ = 8956). Next, the resin was washed with DMF and DCM before deprotecting the Fmoc-group with 20% piperidine in DMF (2 mL, 3 min, 3x). After washing (DMF), Fmoc-Lys(Biotin)-OH (40.4 mg, 68 µmol, 6.8 eq.) dissolved in NMP, PyBOP (35.36 mg, 68 µmol, 6.8 eq.) and DIPEA (26 µL, 150 µmol, 15 eq.) were added. The resin was shaken for 1 hour before a test cleavage confirmed installation of the Lys(biotin) (M + H$^+$ = 9310). Next, the Fmoc-group was deprotected as described before and the final coupling was performed. DiBoc-Rhodamine (28.73 mg, 50 µmol, 5 eq) was added together with PyBOP (26 mg, 50 µmol, 5 eq) and DIPEA (26 µL, 150 µmol, 15 eq) in DMF and after 1 hour of shaking formation of the N-terminus rhodamine and biotin modified ubiquitin was verified (M + H$^+$ = 9445). Subsequently, the resin was washed with DMF and DCM. Alloc deprotection followed by the addition of (Pd(PPH$_3$)$_4$ (2.3 mg, 2 µmol, 0.2 eq) and PhSiH (24.6 µl, 200 µmol, 20 eq) in DCM (2 mL). After shaking for 15 min, the resin was washed with DMF and DCM before repeating the Alloc deprotection procedure twice. Afterwards, a test cleavage confirmed removal of the Alloc group (mass found: (M + H$^+$ = 9361).

*AgNO$_3$ Mediated coupling of Rho-Lys(biotin)-PEG$_2$-Ub$_{1\text{-}76}$(*Arg42Orn) to *compound* ***9***

1.5 µmol Rho-Lys(biotin)-PEG$_2$-Ub$_{1\text{-}76}$ (Arg42Orn) on resin was swelled in DMF before treated with a solution of vinyl-sulfonate isothiourea N-riboside **9** (13 mg, 19.5 µmol, 13 eq.) in DMF (400 µL, 0.05M). Subsequently, Et$_3$N (27 µL, 195 µmol, 130 eq.) and AgNO$_3$ (3.31 mg, 19.5 µmol, 13 eq.) were added and the syringe was wrapped in aluminum foil to protect it from light. The resin was shaken for 2.5 hours before a test cleavage confirmed formation of the conjugate (M + H$^+$ = 9625).

Next, the resin was extensively washed with DMF and DCM before treated with TFA/TIS/H_2O/Phenol (90.5/2/5/2.5, v/v) for 1.5 hours and filtrated in an ice-cold solution of Et_2O:pentane (1:1, v/v). The precipitate formed was centrifuged (5min, 3500 rpm) and the supernatant decanted. The pellet was subsequently dried with N_2, taken up in warm DMSO (1 mL) and diluted with warm water before purified by RP-HPLC. Pure fractions were pooled and lyophilized affording ubiquitin conjugate **5** (320 µg, 0.033 µmol, 2.1%) as a red powder. HRMS: $[C_{429}H_{690}N_{112}O_{134}S_2 + 7H]^{7+}$ found: 1375.9436, calculated: 1376.0014. $[C_{429}H_{690}N_{112}O_{134}S_2 + 8H]^{8+}$ found: 1204.0619, calculated: 1204.1263. $[C_{429}H_{690}N_{112}O_{134}S_2 + 9H]^{9+}$ found: 1070.4370, calculated: 1070.4455. $[C_{429}H_{690}N_{112}O_{134}S_2 + 10H]^{10+}$ found: 963.4996, calculated: 963.5012. $11H]^{11+}$ found: 875.9211, calculated: 876.0001.

HRMS Spectra

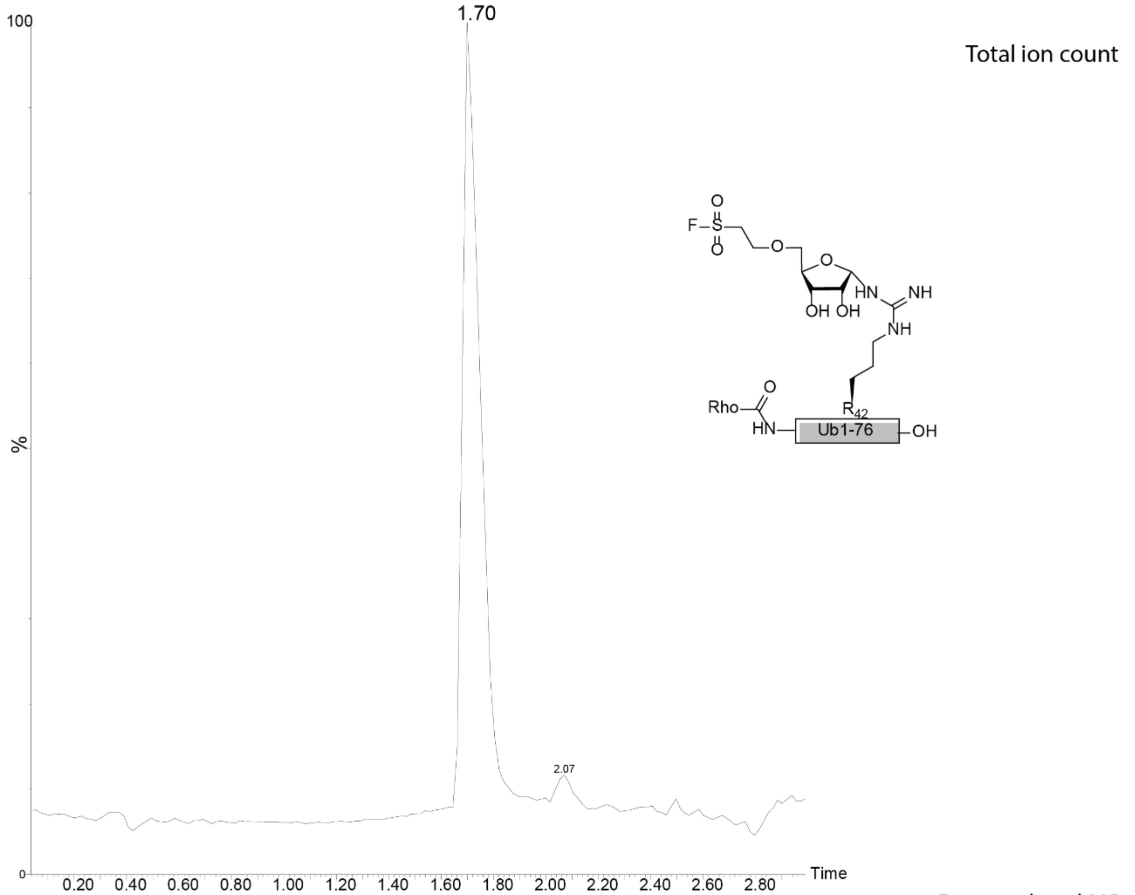

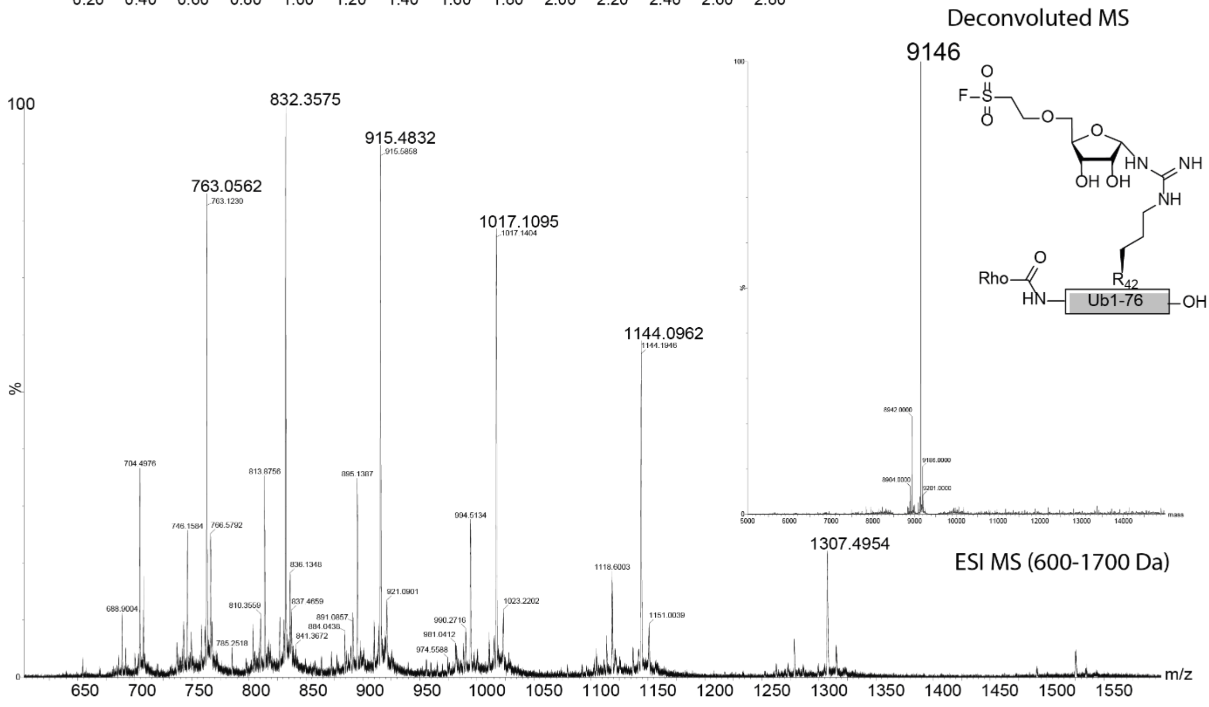

Supplementary Figure 6. HRMS spectra of Probe **1**.

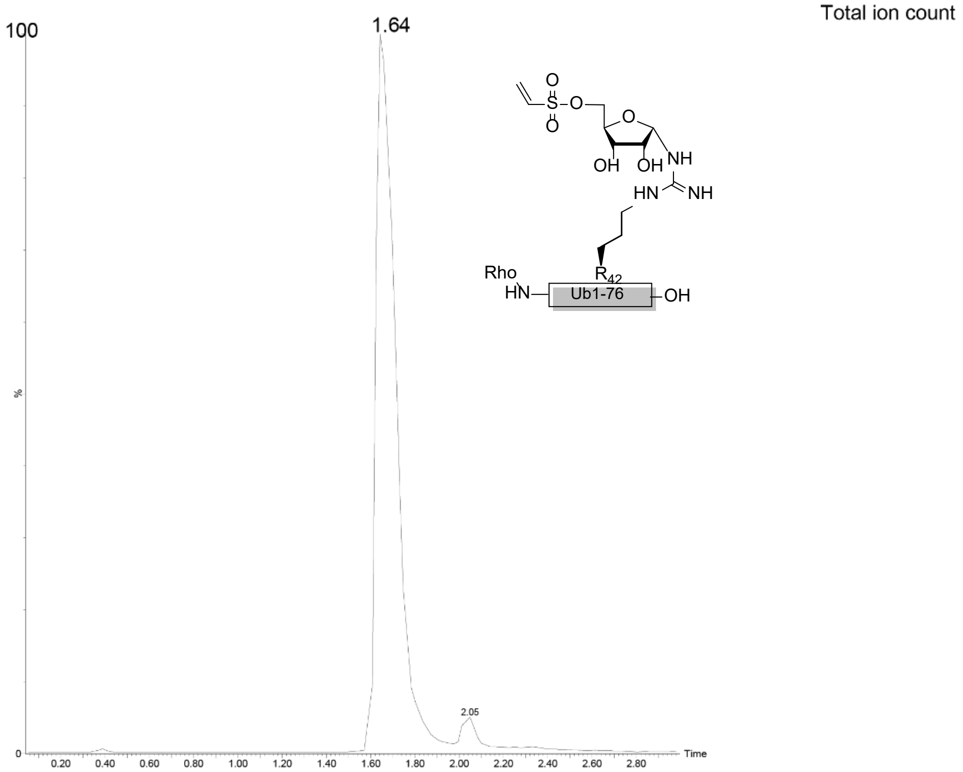

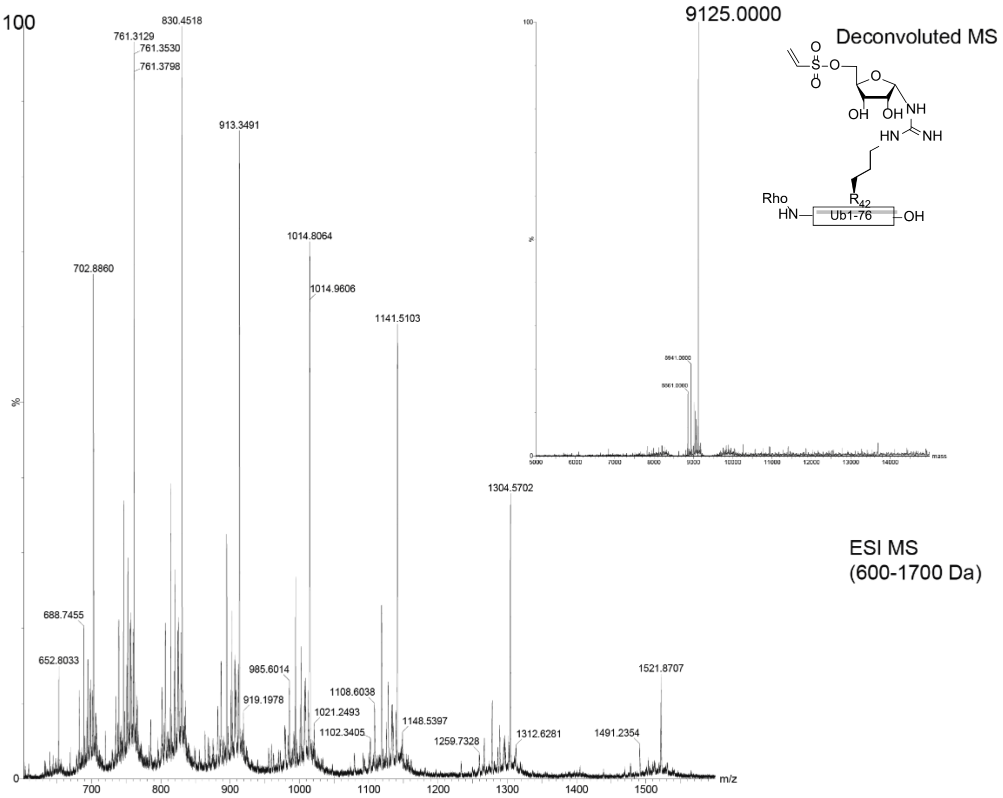

Supplementary Figure 7. HRMS spectra of Probe **2**.

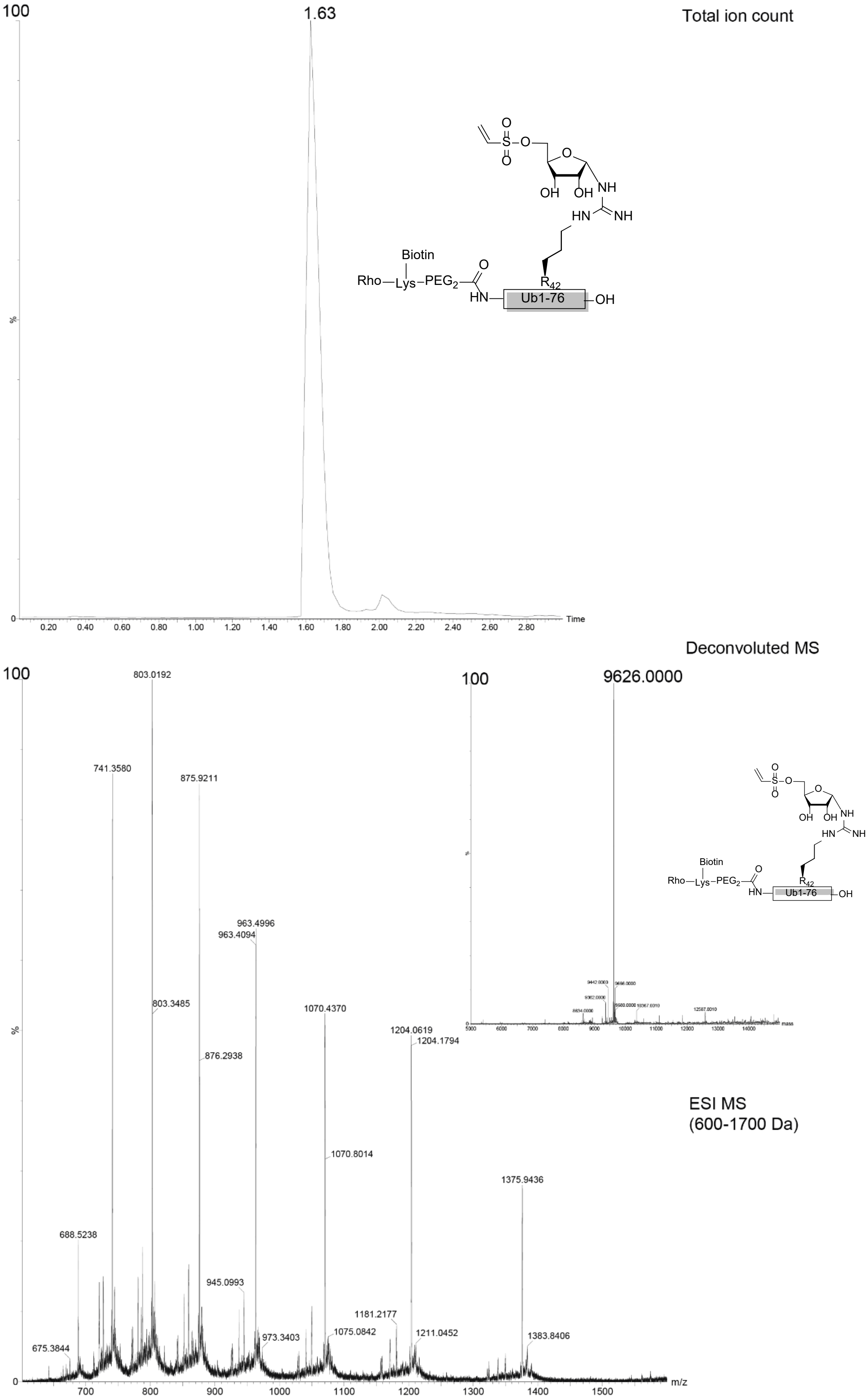

Supplementary Figure 8. HRMS spectra of Probe **5**.

NMR Spectra

^{1}H NMR (**7**)

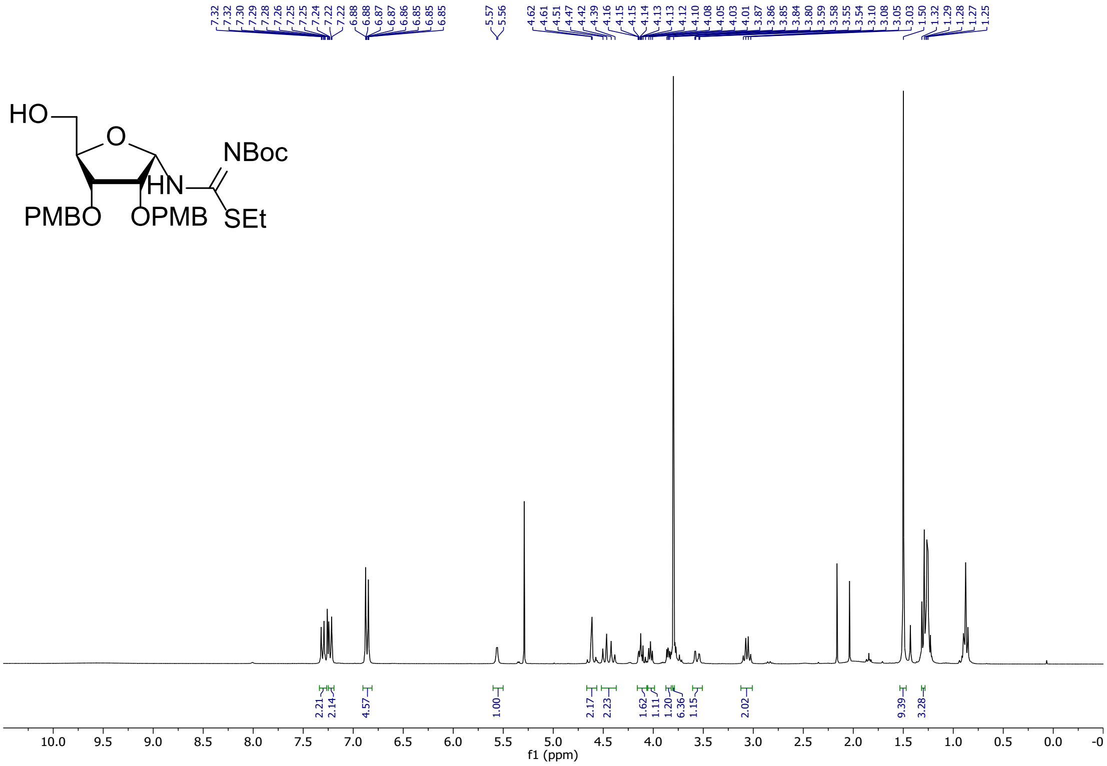

^{13}C NMR (**7**)

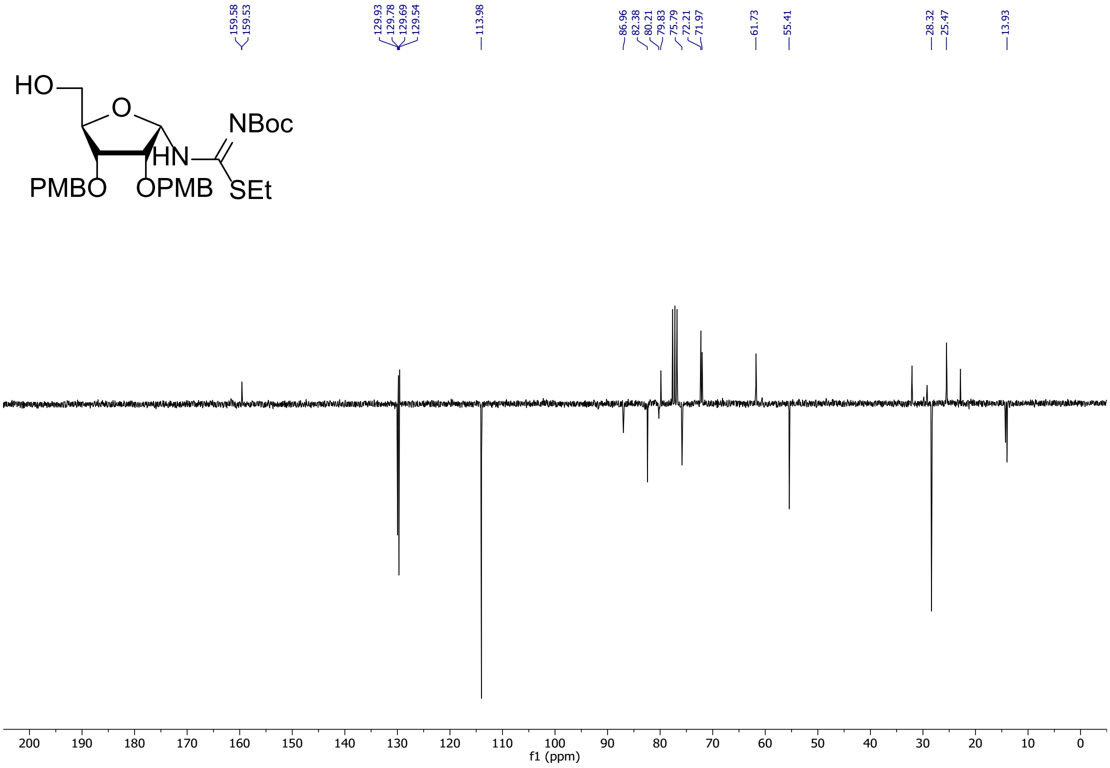

^{1}H NMR (**8**)

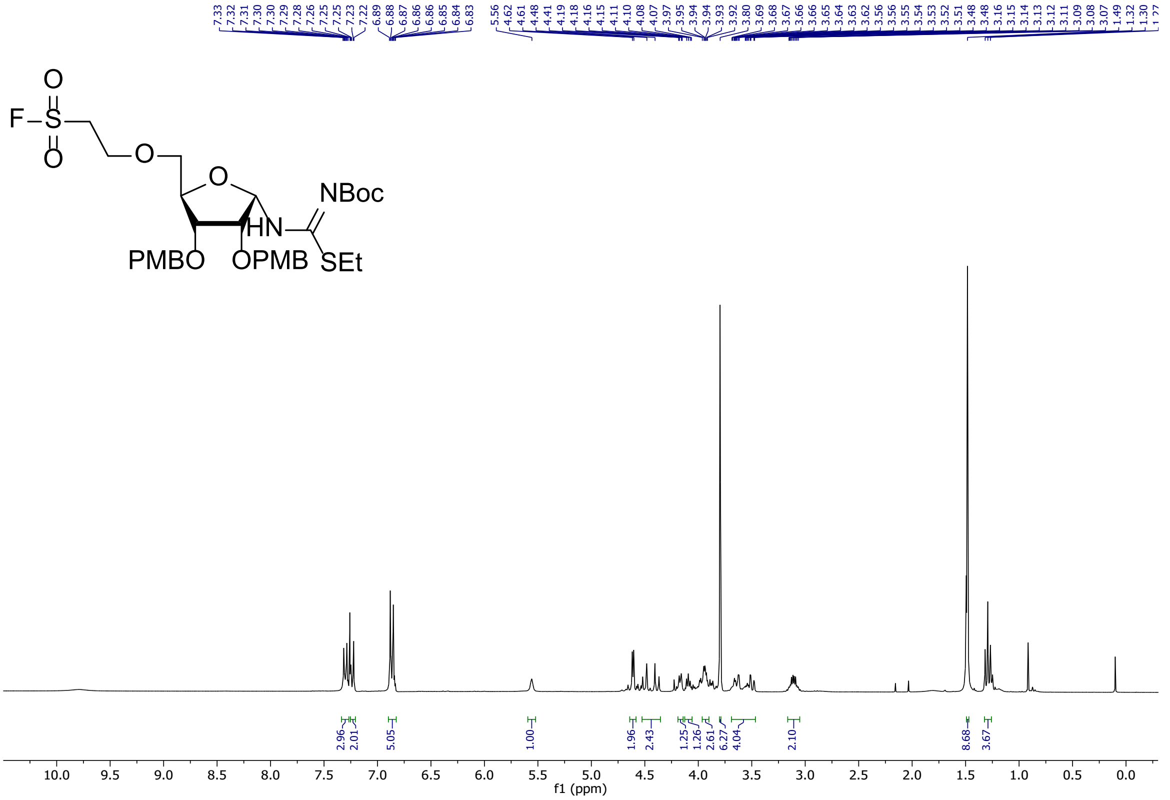

^{13}C NMR (**8**)

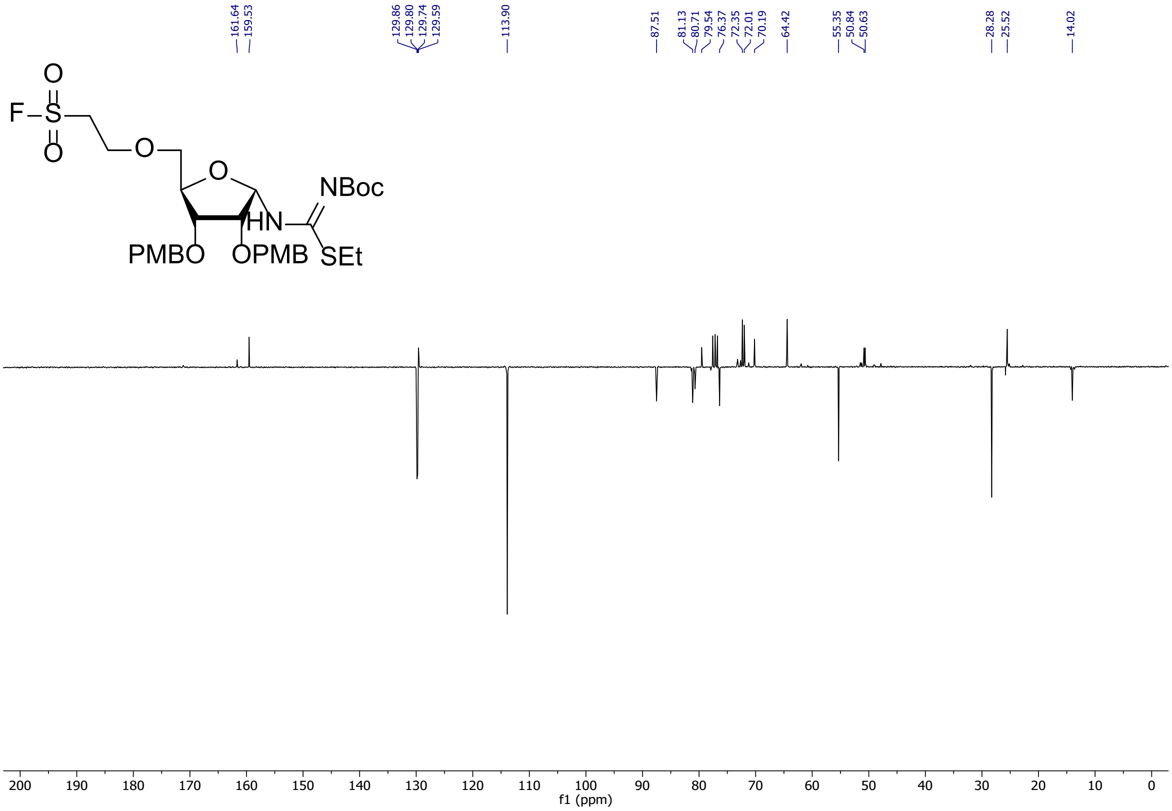

^{19}F NMR (**8**)

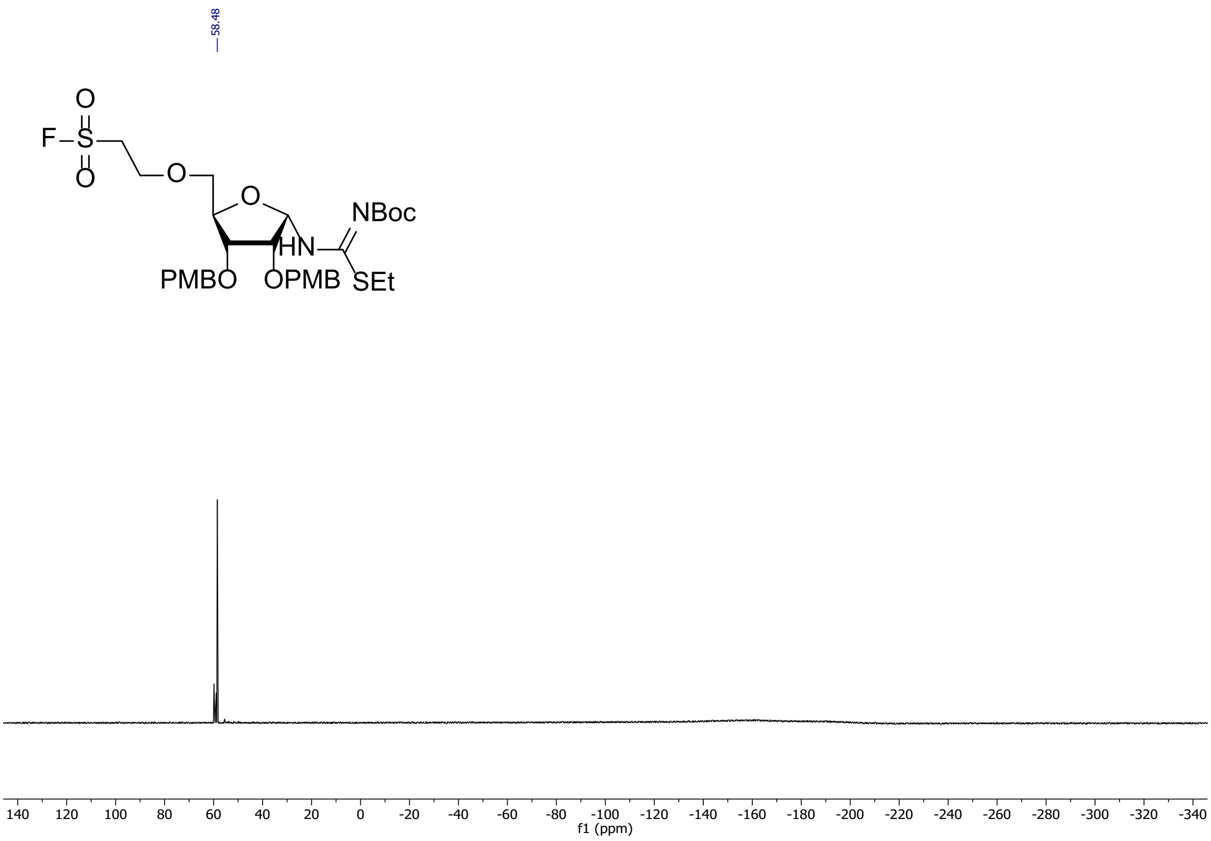

^{1}H NMR (**9**)

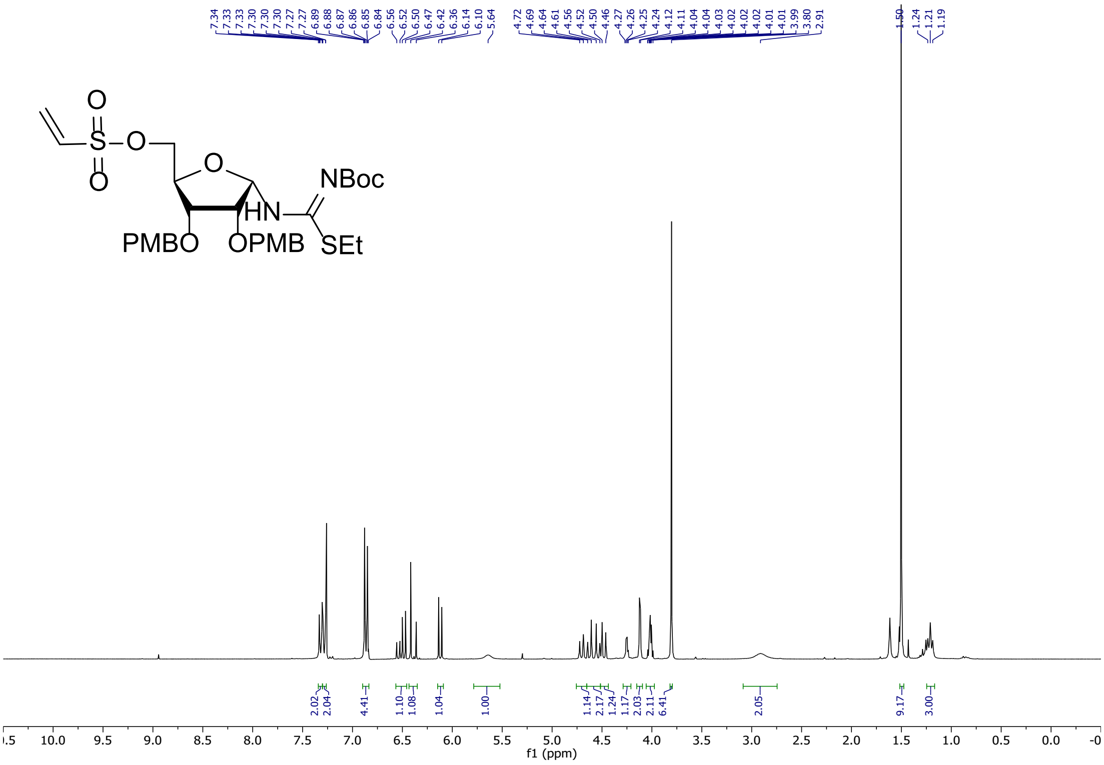

^{13}C NMR (**9**)

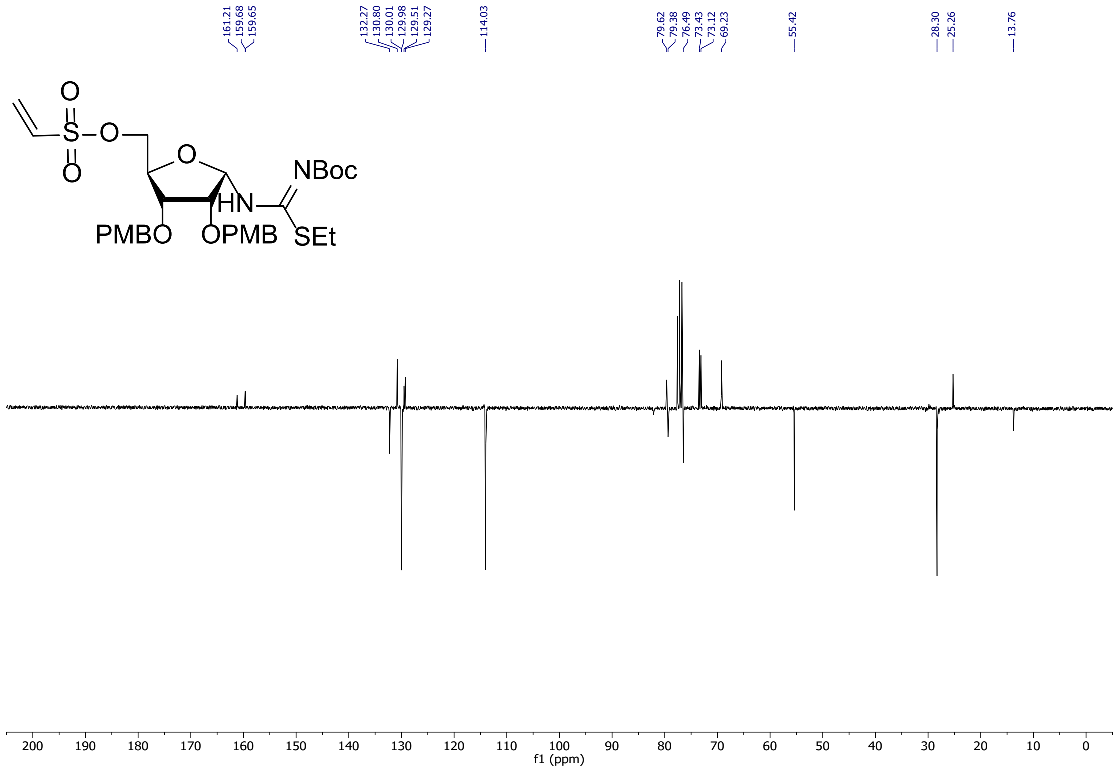

161.21
159.68
159.65
132.27
130.80
130.01
129.98
129.51
129.27
114.03
79.62
79.38
76.49
73.43
73.12
69.23
55.42
28.30
25.26
13.76
O
S
O
O
O
NBoc
HN
PMBO
OPMB
SEt
200 190 180 170 160 150 140 130 120 110 100 90 80 70 60 50 40 30 20 10 0
f1 (ppm)
